# Correction: Genetic diversity of Ethiopian cocoyam (*Xanthosoma sagittifolium* (L.) Schott) accessions as revealed by morphological traits and SSR markers

**DOI:** 10.1371/journal.pone.0253993

**Published:** 2021-06-25

**Authors:** Eyasu Wada, Tileye Feyissa, Kassahun Tesfaye, Zemede Asfaw, Daniel Potter

The first author, Eyasu Wada, is incorrectly noted as the corresponding author. The correct corresponding author is Daniel Potter. Daniel Potter’s email address is: dpotter@ucdavis.edu.

The ORCID iD is missing for the last author. Please see the author’s respective ORCID iDs here:

- Author Daniel Potter’s ORCID iD is: 0000-0002-9855-0355 (https://orcid.org/0000-0002-9855-0355).
