# Supplementary material for: Genetic diversity of Ethiopian cocoyam (Xanthosoma sagittifolium (L.) Schott) accessions as revealed by morphological traits and SSR markers
Source: PLoS One. 2021 Jan 7;16(1):e0245120. doi: 10.1371/journal.pone.0245120 (PMC7790241; doi:10.1371/journal.pone.0245120)
Supplement: S1 Table — (DOCX) [file pone.0245120.s001.docx]

**S1 Table.** **Passport data**: List of cocoyam accessions with accession code, collection sites (Zone, District and Village), coordinate (latitude and longitude) and altitude

| **Accession Code*** | **Zone** | **District** | **Village** | **Latitude (⁰N)** | **Longitude (⁰E)** | **Altitude**  **(masl)** | **Leaf/petiole color** |
| --- | --- | --- | --- | --- | --- | --- | --- |
| BS/Xs001 | Bench-Maji | South-Bench | Kokin | 7.078 | 35.687 | 1594 | Green |
| BS/Xs002 | Bench-Maji | South-Bench | Debrework-01 | 6.891 | 35.703 | 1581 | Green |
| BS/Xs003 | Bench-Maji | South-Bench | Jenehu | 7.023 | 35.651 | 1403 | Green |
| BS/Xs004 | Bench-Maji | South-Bench | Kite | 7.040 | 35.606 | 1345 | Green |
| BS/Xs005 | Bench-Maji | South-Bench | Kite | 7.134 | 35.542 | 1312 | Green |
| BS/Xs006 | Bench-Maji | South-Bench | Kite | 6.939 | 35.714 | 1349 | Green |
| BS/Xs007 | Bench-Maji | South-Bench | Kite | 6.939 | 35.714 | 1349 | Green |
| BN/Xs008 | Bench-Maji | North-Bench | Fanika | 7.129 | 35.571 | 1297 | Green |
| BN/Xs009 | Bench-Maji | North-Bench | Fanika | 7.129 | 35.520 | 1288 | Green |
| BN/Xs010 | Bench-Maji | North-Bench | Woshiken | 7.184 | 35.790 | 1404 | Green |
| BN/Xs011 | Bench-Maji | North-Bench | Woshiken | 7.350 | 35.791 | 1404 | Green |
| BN/Xs012 | Bench-Maji | North-Bench | Temenja-Yazh | 7.210 | 35.791 | 1481 | Green |
| BN/Xs013 | Bench-Maji | North-Bench | Temenja-Yazh | 7.136 | 35.782 | 2070 | Green |
| BN/Xs014 | Bench-Maji | North-Bench | Wacha | 7.137 | 35.781 | 2070 | Green |
| KC/Xs015 | Kefa | Chana | Wacha | 7.373 | 35.919 | 2136 | Green |
| KC/Xs016 | Kefa | Chana | Daha | 7.455 | 36.200 | 1993 | Green |
| KC/Xs017 | Kefa | Chana | Daha | 7.439 | 36.261 | 1913 | Green |
| KC/Xs018 | Kefa | Chana | Woreta | 7.437 | 36.006 | 1810 | Green |
| KC/Xs019 | Kefa | Chana | Woreta | 7.544 | 36.194 | 1800 | Green |
| KG/Xs020 | Kefa | Gimbo | Jakaraba | 7.531 | 36.518 | 1620 | Green |
| KG/Xs021 | Kefa | Gimbo | Ufudo | 7.607 | 36.224 | 1745 | Green |
| KG/Xs022 | Kefa | Gimbo | Ufudo | 7.593 | 36.268 | 1715 | Green |
| KG/Xs023 | Kefa | Gimbo | Shomba-Kichibe | 7.652 | 36.487 | 1445 | Green |
| KG/Xs024 | Kefa | Gimbo | Shomba-Kichibe | 7.476 | 36.518 | 1405 | Green |
| KG/Xs025 | Kefa | Gimbo | Kesh | 7.586 | 36.675 | 1350 | Green |
| KG/Xs026 | Kefa | Gimbo | Jebiye | 7.719 | 36.768 | 1030 | Green |
| DT/Xs027 | Dawuro | Tocha | Gorika-Doma | 7.272 | 37.079 | 1535 | Green |
| DT/Xs028 | Dawuro | Tocha | Gorika-Doma | 7.272 | 37.080 | 1538 | Green |
| DT/Xs029 | Dawuro | Tocha | Wara-Wori | 7.268 | 37.165 | 1357 | Green |
| DT/Xs030 | Dawuro | Tocha | Wara-Wori | 7.354 | 37.071 | 1497 | Green |
| DT/Xs031 | Dawuro | Tocha | Gorika-Dama | 7.289 | 37.186 | 1520 | Green |
| DT/Xs032 | Dawuro | Tocha | Gorika-Dama | 7.333 | 37.016 | 1418 | Green |
| DT/Xs033 | Dawuro | Tocha | Warma-Galcha | 7.246 | 37.259 | 1498 | Green |
| DM/Xs034 | Dawuro | Maraka | Shaba | 7.351 | 37.326 | 1245 | Green |
| DM/Xs035 | Dawuro | Maraka | Shina-Gaburi | 7.294 | 37.312 | 1290 | Green |
| DM/Xs036 | Dawuro | Maraka | Tercha-02 | 7.323 | 37.296 | 1327 | Green |
| DL/Xs037 | Dawuro | Loma | Gasa-Chare | 7.188 | 37.522 | 2129 | Green |
| DL/Xs038 | Dawuro | Loma | Gasa-Chare | 7.124 | 37.533 | 2106 | Green |
| DL/Xs039 | Dawuro | Loma | Gasa-Chare | 7.124 | 37.533 | 2106 | Purple |
| DL/Xs040 | Dawuro | Loma | Gasa-Chare | 7.074 | 37.438 | 2278 | Green |
| DL/Xs041 | Dawuro | Loma | Tulama | 7.054 | 37.345 | 2319 | Green |
| DL/Xs042 | Dawuro | Loma | Tulama | 7.037 | 37.338 | 2299 | Green |
| DL/Xs043 | Dawuro | Loma | Tulama | 7.085 | 37.364 | 2253 | Purple |
| DL/Xs044 | Dawuro | Loma | Elaa-Bacho | 7.160 | 37.426 | 1910 | Green |
| DL/Xs045 | Dawuro | Loma | Elaa-Bacho | 7.170 | 37.426 | 1910 | Purple |
| DB/Xs046 | Dawuro | Gena-Bosa | Lala-Ambe | 7.038 | 37.435 | 1523 | Purple |
| DB/Xs047 | Dawuro | Gena-Bosa | Lala-Ambe | 7.029 | 37.435 | 1523 | Green |
| DB/Xs048 | Dawuro | Gena-Bosa | Deneba | 7.028 | 37.461 | 1221 | Green |
| DB/Xs049 | Dawuro | Gena-Bosa | Deneba | 7.029 | 37.462 | 1221 | Purple |
| **S1 Table.** Continued | | | | | | |  |
| DB/Xs050 | Dawuro | Gena-Bosa | Zima | 7.113 | 37.494 | 1222 | Green |
| DB/Xs051 | Dawuro | Gena-Bosa | Zima | 7.113 | 37.494 | 1222 | Purple |
| DB/Xs052 | Dawuro | Gena-Bosa | Sere-Beta | 7.267 | 37.428 | 1733 | Green |
| DB/Xs053 | Dawuro | Gena-Bosa | Sere-Beta | 7.014 | 37.425 | 1816 | Purple |
| DB/Xs054 | Dawuro | Gena-Bosa | Sere-Beta | 7.269 | 37.425 | 1236 | Green |
| DB/Xs055 | Wolaita | Kindo-Koysha | Bayana | 6.943 | 37.664 | 1132 | Purple |
| WK/Xs056 | Wolaita | Kindo-Koysha | Fagena-Mata | 7.005 | 37.503 | 1160 | Purple |
| WK/Xs057 | Wolaita | Kindo-Koysha | Fagena-Mata | 7.002 | 37.505 | 1158 | Green |
| WK/Xs058 | Wolaita | Kindo-Koysha | Fagena-Mata | 7.077 | 37.712 | 1156 | Purple |
| WK/Xs059 | Wolaita | Kindo-Koysha | Fagena-Mata | 7.078 | 37.714 | 1156 | Green |
| WK/Xs060 | Wolaita | Kindo-Koysha | Bale-01 | 7.115 | 37.660 | 1315 | Purple |
| WK/Xs061 | Wolaita | Kindo-Koysha | Bale-02 | 7.115 | 37.660 | 1315 | Green |
| WH/Xs062 | Wolaita | Humbo | Gututo-Larena | 6.803 | 37.785 | 1849 | Purple |
| WH/Xs063 | Wolaita | Humbo | Gututo-Larena | 7.015 | 38.027 | 1852 | Green |
| WH/Xs064 | Wolaita | Humbo | Gututo-Larena | 6.803 | 37.785 | 1849 | Purple |
| WH/Xs065 | Wolaita | Humbo | Gututo-Larena | 6.858 | 37.942 | 1785 | Green |
| WH/Xs066 | Wolaita | Humbo | Gututo-Larena | 6.858 | 37.942 | 1785 | Purple |
| WH/Xs067 | Wolaita | Humbo | Bosa-Wanche | 6.799 | 37.831 | 1755 | Purple |
| WH/Xs068 | Wolaita | Humbo | Bosa-Wanche | 6.826 | 37.831 | 1746 | Purple |
| WH/Xs069 | Wolaita | Humbo | Demba-Koyisha | 6.768 | 37.767 | 1925 | Purple |
| WS/Xs070 | Wolaita | Sodo-Zuriya | Humbo-Larena | 7.004 | 37.939 | 1836 | Purple |
| WS/Xs071 | Wolaita | Sodo-Zuriya | Wareza-Esho | 7.085 | 37.782 | 1890 | Purple |
| WS/Xs072 | Wolaita | Sodo-Zuriya | Wareza-Esho | 7.085 | 37.782 | 1890 | Green |
| WS/Xs073 | Wolaita | Sodo-Zuriya | Gurumo-Woyde | 7.051 | 37.891 | 1960 | Green |
| WS/Xs074 | Wolaita | Sodo-Zuriya | Wareza-Lasho | 7.085 | 37.931 | 1969 | Green |
| WS/Xs075 | Wolaita | Sodo-Zuriya | Wareza-Lasho | 7.085 | 37.931 | 1969 | Purple |
| WS/Xs076 | Wolaita | Sodo-Zuriya | Damota-Waja | 7.134 | 37.883 | 1940 | Purple |
| WS/Xs077 | Wolaita | Sodo-Zuriya | Damota-Waja | 7.134 | 37.883 | 1940 | Green |
| WB/Xs078 | Wolaita | Boloso-Sore | Dola | 6.187 | 37.798 | 1796 | Purple |
| WB/Xs079 | Wolaita | Boloso-Sore | Dola | 6.268 | 37.923 | 1827 | Purple |
| WB/Xs080 | Wolaita | Boloso-Sore | Gurumo-Koysha | 7.087 | 37.976 | 1914 | Green |
| WB/Xs081 | Wolaita | Boloso-Sore | Gunnuno-01 | 7.146 | 37.875 | 2052 | Purple |
| WB/Xs082 | Wolaita | Boloso-Sore | Gunnuno-02 | 7.119 | 37.892 | 2042 | Purple |
| GQ/Xs083 | Gamo-Gofa | Qucha | Dana Sefera II | 6.697 | 37.678 | 1692 | Green |
| GQ/Xs084 | Gamo-Gofa | Qucha | Dana Sefera II | 6.697 | 37.678 | 1692 | Purple |
| GQ/Xs085 | Gamo-Gofa | Qucha | Basa | 6.578 | 37.675 | 1390 | Green |
| GQ/Xs086 | Gamo-Gofa | Qucha | Basa | 6.578 | 37.675 | 1390 | Purple |
| GQ/Xs087 | Gamo-Gofa | Qucha | Basa | 6.578 | 37.672 | 1393 | Purple |
| GQ/Xs088 | Gamo-Gofa | Qucha | Selamber-03 | 6.583 | 37.461 | 1413 | Green |
| GQ/Xs089 | Gamo-Gofa | Qucha | Selamber-04 | 6.583 | 37.461 | 1413 | Purple |
| GQ/Xs090 | Gamo-Gofa | Qucha | Selamber-01 | 6.639 | 37.704 | 1364 | Green |
| GQ/Xs091 | Gamo-Gofa | Qucha | Selamber-02 | 6.639 | 37.704 | 1364 | Purple |
| GQ/Xs092 | Gamo-Gofa | Qucha | Morka | 6.706 | 37.667 | 1325 | Purple |
| GQ/Xs093 | Gamo-Gofa | Qucha | Morka | 6.448 | 37.375 | 1314 | Purple |
| GQ/Xs094 | Gamo-Gofa | Qucha | Morka | 6.448 | 37.375 | 1314 | Green |
| GD/Xs095 | Gamo-Gofa | Daramalo | Dita | 6.676 | 37.508 | 1204 | Purple |
| GD/Xs096 | Gamo-Gofa | Demba-Gofa | Dorga | 6.458 | 36.983 | 1192 | Purple |
| GD/Xs097 | Gamo-Gofa | Demba-Gofa | Dorga | 6.464 | 37.073 | 1816 | Green |
| GD/Xs098 | Gamo-Gofa | Demba-Gofa | Dorga | 6.417 | 37.056 | 1189 | Green |
| GD/Xs099 | Gamo-Gofa | Demba-Gofa | Boreda | 6.486 | 36.947 | 1306 | Purple |
| GD/Xs100 | Gamo-Gofa | Demba-Gofa | Boreda | 6.486 | 36.947 | 1306 | Green |

*In the accession code the first two letters stand for the Zone and District of where an acccession was collected followed by Xs for species name and serial number of accessions.
