# Supplementary material for: Genetic diversity of Ethiopian cocoyam (Xanthosoma sagittifolium (L.) Schott) accessions as revealed by morphological traits and SSR markers
Source: PLoS One. 2021 Jan 7;16(1):e0245120. doi: 10.1371/journal.pone.0245120 (PMC7790241; doi:10.1371/journal.pone.0245120)
Supplement: S2 Table — (DOCX) [file pone.0245120.s002.docx]

| **S2 Table. Selected morphological descriptors used to characterize cocoyam (*Xanthosoma sagittifolium*) grown in Ethiopia** | | |
| --- | --- | --- |
| **S. N** | **Qualitative character** | **Character state** |
| 1 | Plant growth habit | 1. Acaulescent 2. Erect above ground stem 3. Reclining aboveground stem |
| 2 | Petiole attachment | 1. Peltate 2. Subpeltate 3. Non peltate |
| 3 | Petiole color (upper 2/3^rd^) | 1. Light green 2. Green 3. Red/Purple 4. Green streaked with red/purple |
| 4 | Petiole color (lower 1/3^rd^) | 1. Light green 2 Green 3. Red/Purple 4. Green streaked with red/purple |
| 5 | Color of edge of petiole sheath | 1. The same as the rest of petiole and sheath 3. Lighter than the rest of petiole and sheath  2. Darker than the rest of petiole and sheath 4. Pink/Red/Purple |
| 6 | Lamina orientation | 1. One plan - apex up (Erect) 2. One plane - apex down (Droopy) 3. 3-dimentional (cup shaped) |
| 7 | Leaf margin color | 1. Green to the edge 2. Clear to the edge 3. Purple/Red edge 4. Pale yellow/creamy edge |
| 8 | Leaf shape | 1. No basal lobes 2. Sagittate- basal lobes >1/8^th^ -1/4^th^ the length of leaf 3. Hastate (basal lobes flared)  4. Sagittate-basal lobes <1/8^th^ the length of leaf 5. Sagittate basal lobes >1/4^th^ the length of leaf |
| 9 | Color of upper leaf surface | 1. Light green 2. Medium green 3. Dark green 4. Redish/ Purplish green 5. Other |
| 10 | Color of lower leaf surface | 1. Light green 2. Medium green 3. Dark green 4. Redish/ Purplish green 5. Other |
| 11 | Color of veins on upper leaf surface | 1. Same as color as lamina 3. Lighter green than lamina  2. Darker green than lamina 4. Red/Purple |
| 12 | Color of veins on lower leaf surface | 1. Same as color as lamina 2. Darker green than lamina 3. Lighter green than lamina 4. Red/Purple |
| 13 | Position of cormel apex | 1. Aboveground 2. Underground 3. Both |
| 14 | Shape of cormels | 1. Globose 2. Ovate 3. Cylindrical 4. Elliptical 5. Mixed (state which of these---) |
| 15 | Color of cormel apex | 1. White 2. Pink 3. Red |
| 16 | Flesh cormel color | 1. White 2. Yellow 3. Orange 4. Pink or pale red 5. Purple |
| **Quantitative trait** | | **Remark** |
| 1 | Over all plant height (cm | Measured from ground level to the top of plant |
| 2 | Petiole length (cm) | Length of the longest petiole from the basal zone of the plant to the point of leaf attachment |
| 3 | Petiole sheath length (cm | Length of the sheath of longest petiole from the beginning to the end to sheath |
| 4 | Leaf length (cm) | The length of the leaf was measured |
| 5 | Leaf width (cm) | The width of the leaf was measured |
| 6 | Circumference of the above ground stem (cm) | The circumference of pseudostem just above ground measured |
| 7 | Number of cormels | The cormels produced by a plant counted |
| 8 | Cormel length (cm) | The length of large. medium and small sized cormels measured |
| 9 | Cormel diameter (cm | The diameter of large. medium and small sized cormels measured |
| 10 | Cormel fresh weight per plant (kg) | The weight of cormels per plant weighed |
| 11 | Corm length (cm) | The length of corm measured |
| 12 | Corm diameter (cm) | The diameter of corm measured |
| 13 | Corm fresh weight (kg) | The fresh weight of corm per plant was weighed |
