# Supplementary material for: Genetic diversity of Ethiopian cocoyam (Xanthosoma sagittifolium (L.) Schott) accessions as revealed by morphological traits and SSR markers
Source: PLoS One. 2021 Jan 7;16(1):e0245120. doi: 10.1371/journal.pone.0245120 (PMC7790241; doi:10.1371/journal.pone.0245120)
Supplement: S3 Table — (DOCX) [file pone.0245120.s003.docx]

| **S3 Table. Primers sequences used for amplification of microsatellite markers.** | | | | |
| --- | --- | --- | --- | --- |
| **Duplex** | **Locus name** | **Repeat motif** | **Primer sequence (5 ′-3 ′)** | **Primer direction** |
| A | mXsCIR05 | (CA)8 (CACA)3 | 6-FAM-CGCATTATTAACGAATATC | Forward |
|  |  |  | TCATCTATGGCTATCACCT | Reverse |
|  | mXsCIR07 | (TG)7 (AG)19 | HEX -GGACTGGGAGTCTGAGTAG | Forward |
|  |  |  | CCTTTCCCCTCACTATAAA | Reverse |
| B | mXsCIR10 | (AG)22 | 6-FAM-ATGTCTGTAGTGGCCTAGT | Forward |
|  |  |  | AATTAAGTTGGGTGGTAGAT | Reverse |
|  | mXsCIR22 | (AG)22 | HEX-CGTGAGAAACACCTGAATTA | Forward |
|  |  |  | AATTTGCTCTGTCATTGTG | Reverse |
| C | mXsCIR11 | (TG)10 (GA)16 | 6-FAM-AATTCTTAGCAGCATTGTTA | Forward |
|  |  |  | CATTCGTATCAACTTCCTTT | Reverse |
|  | mXsCIR24 | (AG)23 | HEX- AATTTGAAGTGAAACGATCA | Forward |
|  |  |  | TCCTGTCATCAGAATTGTA | Reverse |
| D | mXsCIR12 | (TC)17 (TTC)7 (TCCC)3 (TTCTTG)3 | 6-FAM-TACATTTCCATTGCCATC | Forward |
|  |  |  | AAATTAAAGAGGGAGACAG | Reverse |
|  | mXsCIR27 | (AG)15 (GAA)6 | HEX-TGCATGAATTGAAGAAAT | Forward |
|  |  |  | AACAAAGAGTCTCACCACAT | Reverse |
| E | mXsCIR19 | (AC)8 (AC)24 (AC)8 | 6-FAM -AACTTGTGTATCCTACATCC | Forward |
|  |  |  | GCGTGGTTTATGTGTATCTT | Reverse |
|  | mXsCIR21 | (AG)30 | HEX-CTTAACCTTGTCAGCCTCT | Forward |
|  |  |  | GAGCGGTATAACAATTCATC | Reverse |
| F | mXsCIR16 | (AG)15 | 6-FAM-CTTATTGATGCCGAGAATAC | Forward |
|  |  |  | TTCCTCACAATATGTTCTCAT | Reverse |
|  | mXsCIR28 | (GA)9 | HEX- ACAGAAGTTGACATGGAGAG | Forward |
|  |  |  | AATGTTAAAGAGCAAAAGGA | Reverse |
