# Supplementary material for: Genetic diversity of Ethiopian cocoyam (Xanthosoma sagittifolium (L.) Schott) accessions as revealed by morphological traits and SSR markers
Source: PLoS One. 2021 Jan 7;16(1):e0245120. doi: 10.1371/journal.pone.0245120 (PMC7790241; doi:10.1371/journal.pone.0245120)
Supplement: S4 Table — (DOCX) [file pone.0245120.s004.docx]

**S4 Table.** **Mean performances of 13 quantitative traits of 100 cocoyam accessions**: Cormel (100) was planted in a 10*10 simple lattice square design in the 2016 cropping season. The experiment was also repeated in the 2017 cropping season using the same experimental design and similar crop management practices.

| **Accessions’ code** | **Morphotype** | **PH** | **PL** | **PSL** | **LL** | **LW** | **CAGS** | **NC** | **CL** | **CD** | **CFW** | **CrL** | **CrD** | **CrFw** |
| --- | --- | --- | --- | --- | --- | --- | --- | --- | --- | --- | --- | --- | --- | --- |
| BS/Xs001 | Green | 78.25 | 58.67 | 31.08 | 42.78 | 24.89 | 32.25 | 12.83 | 9.42 | 3.38 | 1.90 | 11.83 | 6.21 | 1.25 |
| BS/Xs002 | Green | 78.08 | 62.00 | 33.92 | 41.56 | 23.67 | 31.75 | 11.83 | 9.00 | 3.10 | 1.40 | 12.08 | 5.73 | 1.20 |
| BS/Xs003 | Green | 81.33 | 63.33 | 33.83 | 36.42 | 21.31 | 29.83 | 10.25 | 9.08 | 2.56 | 1.25 | 11.50 | 5.24 | 0.93 |
| BS/Xs004 | Green | 72.42 | 56.33 | 29.50 | 35.06 | 19.67 | 25.75 | 11.83 | 9.00 | 2.85 | 1.33 | 12.33 | 5.58 | 1.25 |
| BS/Xs005 | Green | 83.58 | 64.58 | 39.25 | 43.17 | 24.11 | 32.33 | 15.33 | 9.75 | 2.93 | 1.88 | 13.17 | 6.96 | 1.23 |
| BS/Xs006 | Green | 84.83 | 67.42 | 41.42 | 36.81 | 21.50 | 33.00 | 9.58 | 11.18 | 3.10 | 1.28 | 13.50 | 6.31 | 0.98 |
| BS/Xs007 | Green | 72.33 | 54.17 | 30.42 | 38.92 | 23.08 | 28.08 | 12.08 | 10.83 | 3.04 | 1.85 | 11.42 | 5.75 | 1.03 |
| BN/Xs008 | Green | 80.92 | 53.83 | 36.08 | 40.25 | 22.89 | 33.82 | 13.33 | 10.58 | 3.98 | 2.43 | 11.33 | 6.64 | 1.63 |
| BN/Xs009 | Green | 74.42 | 55.50 | 32.67 | 35.86 | 20.53 | 24.17 | 10.08 | 8.92 | 3.48 | 1.38 | 10.33 | 5.38 | 0.98 |
| BN/Xs010 | Green | 75.25 | 55.83 | 34.33 | 36.14 | 23.22 | 27.50 | 8.25 | 9.08 | 2.25 | 0.90 | 10.50 | 4.79 | 2.10 |
| BN/Xs011 | Green | 79.08 | 65.58 | 38.75 | 39.56 | 22.56 | 30.92 | 11.33 | 8.92 | 3.23 | 1.10 | 12.92 | 6.27 | 1.73 |
| BN/Xs012 | Green | 67.92 | 52.25 | 28.50 | 35.78 | 20.89 | 28.08 | 7.33 | 7.08 | 2.68 | 1.08 | 11.33 | 4.66 | 0.88 |
| BN/Xs013 | Green | 66.83 | 52.58 | 28.75 | 32.97 | 20.31 | 26.17 | 10.33 | 8.33 | 2.41 | 1.15 | 9.33 | 5.08 | 0.65 |
| BN/Xs014 | Green | 73.67 | 53.42 | 30.67 | 33.19 | 19.94 | 25.17 | 9.92 | 9.83 | 2.96 | 1.35 | 10.25 | 5.23 | 0.88 |
| KC/Xs015 | Green | 68.17 | 48.92 | 28.33 | 31.11 | 19.14 | 25.08 | 9.75 | 10.33 | 2.59 | 1.13 | 10.83 | 5.73 | 0.90 |
| KC/Xs016 | Green | 66.25 | 51.50 | 27.92 | 35.47 | 20.19 | 27.25 | 12.67 | 9.50 | 3.20 | 1.35 | 13.08 | 5.49 | 0.95 |
| KC/Xs017 | Green | 75.58 | 55.25 | 33.33 | 38.06 | 22.75 | 28.83 | 11.75 | 10.67 | 3.35 | 1.45 | 12.50 | 5.88 | 1.18 |
| KC/Xs018 | Green | 72.83 | 53.17 | 29.92 | 33.69 | 20.81 | 27.58 | 11.17 | 10.33 | 3.09 | 1.48 | 11.83 | 5.49 | 1.10 |
| KC/Xs019 | Green | 74.08 | 54.33 | 30.17 | 38.11 | 19.92 | 29.08 | 12.67 | 9.50 | 3.22 | 1.30 | 11.71 | 5.59 | 0.78 |
| KG/Xs020 | Green | 68.17 | 50.17 | 28.50 | 29.69 | 17.28 | 25.42 | 5.92 | 6.58 | 2.69 | 0.68 | 7.42 | 3.49 | 0.58 |
| KG/Xs021 | Green | 66.08 | 50.00 | 26.25 | 36.25 | 20.47 | 28.00 | 12.67 | 6.75 | 2.69 | 0.98 | 10.83 | 5.39 | 1.15 |
| KG/Xs022 | Green | 70.50 | 56.92 | 31.25 | 36.69 | 21.03 | 27.00 | 11.08 | 8.83 | 2.86 | 1.45 | 10.50 | 5.81 | 0.90 |
| KG/Xs023 | Green | 75.33 | 58.58 | 33.92 | 33.33 | 20.00 | 26.33 | 9.75 | 7.42 | 2.64 | 1.13 | 12.58 | 5.18 | 0.98 |
| KG/Xs024 | Green | 65.00 | 45.92 | 30.33 | 30.53 | 18.42 | 23.50 | 8.83 | 7.17 | 2.42 | 0.88 | 9.92 | 4.48 | 1.03 |
| KG/Xs025 | Green | 74.67 | 56.33 | 31.17 | 35.11 | 20.86 | 27.92 | 9.08 | 10.25 | 2.80 | 1.03 | 12.00 | 5.35 | 0.83 |
| KG/Xs026 | Green | 71.33 | 52.58 | 30.25 | 39.08 | 22.97 | 29.08 | 13.00 | 11.83 | 3.00 | 1.60 | 10.00 | 5.70 | 1.53 |
| KC/Xs027 | Green | 76.25 | 55.67 | 30.50 | 38.17 | 23.36 | 29.00 | 9.25 | 9.67 | 2.79 | 1.00 | 11.92 | 4.96 | 0.83 |
| **S4 Table Continued** | | | | | | | | | | | | | | |
| DT/Xs028 | Green | 69.42 | 51.75 | 29.67 | 34.44 | 22.39 | 25.92 | 11.58 | 9.75 | 2.78 | 1.75 | 9.75 | 4.79 | 1.15 |
| DT/Xs029 | Green | 77.17 | 58.58 | 36.17 | 35.03 | 20.44 | 27.33 | 13.17 | 8.58 | 3.11 | 1.48 | 10.50 | 6.12 | 1.00 |
| DT/Xs030 | Green | 74.25 | 51.92 | 30.58 | 37.17 | 21.58 | 28.75 | 6.75 | 8.42 | 2.61 | 0.90 | 6.08 | 3.81 | 0.53 |
| DT/Xs031 | Green | 75.50 | 57.83 | 33.08 | 36.06 | 21.22 | 33.33 | 10.75 | 8.42 | 3.05 | 0.90 | 12.00 | 5.93 | 1.05 |
| DT/Xs032 | Green | 77.00 | 58.50 | 34.83 | 37.47 | 23.06 | 31.75 | 11.42 | 10.00 | 3.16 | 1.18 | 11.92 | 6.15 | 1.13 |
| DT/Xs033 | Green | 67.17 | 50.25 | 28.08 | 35.47 | 22.14 | 27.67 | 8.42 | 8.33 | 3.81 | 1.40 | 11.50 | 6.28 | 0.98 |
| DM/Xs034 | Green | 68.42 | 49.33 | 27.33 | 36.89 | 21.89 | 26.67 | 8.92 | 8.08 | 3.18 | 0.90 | 10.92 | 5.98 | 1.20 |
| DM/Xs035 | Green | 67.75 | 56.50 | 30.58 | 35.00 | 19.97 | 27.58 | 10.17 | 9.42 | 2.79 | 0.83 | 11.58 | 5.12 | 0.90 |
| DM/Xs036 | Green | 71.67 | 55.08 | 31.58 | 39.64 | 23.06 | 29.50 | 10.83 | 8.83 | 3.32 | 1.18 | 10.92 | 6.01 | 0.85 |
| DL/Xs037 | Green | 74.00 | 55.33 | 29.42 | 40.28 | 24.50 | 31.50 | 11.25 | 9.50 | 2.76 | 1.15 | 13.17 | 6.05 | 0.83 |
| DL/Xs038 | Green | 70.67 | 48.33 | 29.58 | 34.06 | 20.33 | 26.83 | 10.17 | 8.58 | 2.39 | 1.35 | 10.67 | 5.27 | 1.23 |
| DL/Xs039 | Purple | 77.42 | 60.58 | 32.92 | 38.61 | 22.58 | 29.83 | 11.33 | 9.50 | 3.79 | 1.38 | 11.42 | 5.98 | 1.63 |
| DL/Xs040 | Green | 77.83 | 56.25 | 33.83 | 39.25 | 23.33 | 31.33 | 9.50 | 9.08 | 3.17 | 1.30 | 8.42 | 5.38 | 0.90 |
| DL/Xs041 | Green | 66.08 | 51.58 | 27.92 | 35.06 | 19.78 | 25.33 | 8.83 | 7.58 | 2.60 | 1.13 | 9.00 | 4.29 | 1.00 |
| DL/Xs042 | Green | 73.00 | 54.75 | 31.08 | 34.33 | 20.75 | 27.92 | 6.92 | 8.17 | 3.50 | 0.95 | 9.83 | 5.16 | 0.90 |
| DL/Xs043 | Purple | 66.58 | 54.58 | 30.75 | 40.89 | 22.69 | 28.25 | 8.75 | 9.50 | 3.46 | 1.95 | 13.25 | 6.46 | 1.73 |
| DL/Xs044 | Green | 69.25 | 52.17 | 29.83 | 38.11 | 22.14 | 27.67 | 11.42 | 8.17 | 3.16 | 1.58 | 10.00 | 5.78 | 1.05 |
| DL/Xs045 | Purple | 69.33 | 59.33 | 30.33 | 43.11 | 23.11 | 32.25 | 7.58 | 7.42 | 3.53 | 1.00 | 16.58 | 6.48 | 2.30 |
| DB/Xs046 | Purple | 64.75 | 53.08 | 29.83 | 40.28 | 22.50 | 34.42 | 11.08 | 8.83 | 3.25 | 1.25 | 13.75 | 5.53 | 1.68 |
| DB/Xs047 | Green | 68.50 | 50.92 | 28.58 | 36.08 | 20.33 | 28.83 | 9.42 | 10.33 | 2.62 | 0.75 | 12.75 | 5.54 | 0.90 |
| DB/Xs048 | Green | 68.33 | 49.33 | 27.75 | 37.17 | 23.56 | 27.58 | 9.67 | 9.33 | 3.02 | 0.80 | 10.50 | 5.00 | 1.18 |
| DB/Xs049 | Purple | 71.59 | 53.83 | 29.75 | 38.17 | 23.39 | 29.08 | 9.50 | 9.50 | 3.68 | 1.38 | 12.92 | 6.37 | 1.23 |
| DB/Xs050 | Green | 71.92 | 50.08 | 27.92 | 35.75 | 21.86 | 25.83 | 8.42 | 9.75 | 2.90 | 0.90 | 8.75 | 4.41 | 0.83 |
| DB/Xs051 | Purple | 67.09 | 55.50 | 29.00 | 38.31 | 22.83 | 31.25 | 8.92 | 8.42 | 3.18 | 1.00 | 14.08 | 6.10 | 1.05 |
| DB/Xs052 | Green | 73.83 | 54.42 | 30.42 | 35.53 | 21.03 | 26.83 | 10.42 | 9.08 | 3.25 | 1.43 | 11.33 | 5.65 | 1.33 |
| DB/Xs053 | Purple | 65.75 | 58.92 | 38.33 | 45.25 | 24.25 | 30.42 | 8.67 | 7.17 | 3.23 | 0.95 | 13.08 | 5.31 | 1.50 |
| DB/Xs054 | Green | 70.25 | 53.33 | 25.50 | 35.47 | 20.94 | 27.08 | 8.17 | 7.92 | 2.87 | 1.10 | 10.92 | 5.63 | 1.08 |
| DB/Xs055 | Purple | 64.75 | 52.83 | 30.67 | 37.50 | 22.06 | 27.75 | 8.58 | 8.08 | 3.68 | 1.20 | 15.92 | 5.70 | 1.15 |
| WK/Xs056 | Purple | 71.33 | 54.33 | 29.42 | 38.89 | 22.33 | 29.25 | 9.67 | 10.25 | 3.79 | 1.08 | 11.17 | 6.97 | 1.68 |
| WK/Xs057 | Green | 75.50 | 56.33 | 27.58 | 38.14 | 23.08 | 29.83 | 9.58 | 9.42 | 3.03 | 1.08 | 11.33 | 5.98 | 0.78 |
| **S4 Table Continued** | | | | | | | | | | | | | | |
| WK/Xs058 | Purple | 62.59 | 51.08 | 29.42 | 35.67 | 20.53 | 27.58 | 8.92 | 10.67 | 3.90 | 1.25 | 13.92 | 6.17 | 1.48 |
| WK/Xs059 | Green | 62.33 | 44.67 | 26.25 | 37.92 | 22.25 | 28.00 | 8.67 | 10.50 | 2.88 | 0.93 | 8.92 | 5.70 | 0.88 |
| WK/Xs060 | Purple | 62.00 | 49.83 | 26.17 | 33.72 | 19.92 | 25.00 | 10.17 | 8.75 | 3.48 | 1.13 | 11.92 | 5.33 | 1.23 |
| WK/Xs061 | Green | 69.08 | 48.08 | 27.92 | 36.53 | 22.47 | 29.00 | 9.17 | 9.17 | 2.94 | 1.28 | 9.67 | 4.90 | 1.10 |
| WH/Xs062 | Purple | 74.59 | 57.92 | 33.08 | 40.06 | 25.47 | 30.92 | 11.25 | 8.25 | 3.36 | 1.63 | 13.83 | 5.76 | 1.00 |
| WH/Xs063 | Green | 75.67 | 58.67 | 32.17 | 35.94 | 21.42 | 25.75 | 9.17 | 9.00 | 2.96 | 1.23 | 10.33 | 5.48 | 1.05 |
| WH/Xs064 | Purple | 70.67 | 56.58 | 31.67 | 40.39 | 22.50 | 28.17 | 8.67 | 8.33 | 3.66 | 1.10 | 14.50 | 5.66 | 1.28 |
| WH/Xs065 | Green | 63.00 | 50.08 | 28.83 | 32.58 | 18.11 | 24.50 | 9.75 | 8.08 | 3.03 | 1.13 | 11.25 | 3.78 | 0.75 |
| WH/Xs066 | Purple | 67.08 | 55.08 | 33.50 | 37.42 | 20.92 | 30.25 | 12.58 | 8.58 | 4.21 | 1.55 | 16.08 | 6.43 | 1.35 |
| WH/Xs067 | Purple | 67.67 | 54.75 | 30.75 | 40.89 | 22.67 | 31.08 | 9.58 | 8.42 | 3.54 | 1.15 | 14.50 | 6.95 | 1.85 |
| WH/Xs068 | Purple | 57.58 | 42.75 | 25.50 | 38.22 | 21.83 | 31.83 | 11.67 | 8.75 | 3.80 | 1.48 | 12.42 | 6.22 | 1.65 |
| WH/Xs069 | Purple | 71.42 | 55.58 | 31.83 | 38.83 | 21.14 | 28.17 | 9.00 | 9.25 | 4.11 | 1.20 | 12.75 | 6.15 | 1.65 |
| WS/Xs070 | Purple | 78.25 | 63.50 | 36.33 | 42.33 | 24.39 | 31.33 | 8.92 | 9.50 | 3.93 | 1.48 | 12.00 | 7.28 | 1.68 |
| WS/Xs071 | Purple | 65.92 | 55.58 | 28.50 | 37.64 | 22.44 | 32.92 | 11.67 | 8.58 | 3.72 | 1.73 | 13.92 | 5.99 | 1.65 |
| WS/Xs072 | Green | 71.50 | 51.58 | 31.58 | 40.75 | 24.19 | 30.25 | 13.50 | 11.25 | 3.14 | 1.75 | 10.08 | 5.68 | 0.85 |
| WS/Xs073 | Green | 75.67 | 53.50 | 33.50 | 36.22 | 23.50 | 24.50 | 12.92 | 8.42 | 3.13 | 1.63 | 8.92 | 5.50 | 1.08 |
| WS/Xs074 | Green | 75.08 | 55.17 | 31.00 | 38.06 | 23.33 | 27.92 | 10.00 | 10.00 | 3.53 | 1.48 | 10.67 | 5.33 | 1.08 |
| WS/Xs075 | Purple | 74.75 | 64.25 | 36.08 | 39.28 | 22.33 | 30.08 | 8.67 | 8.92 | 3.38 | 1.15 | 13.17 | 5.40 | 1.05 |
| WS/Xs076 | Purple | 80.17 | 66.50 | 43.92 | 43.33 | 23.69 | 30.08 | 8.33 | 9.50 | 3.95 | 1.20 | 14.17 | 6.51 | 1.63 |
| WS/Xs077 | Green | 76.08 | 53.25 | 29.83 | 38.44 | 22.17 | 27.75 | 11.33 | 8.50 | 3.26 | 1.50 | 11.17 | 5.63 | 0.93 |
| WB/Xs078 | Purple | 72.50 | 59.75 | 32.75 | 40.44 | 22.67 | 30.92 | 8.00 | 8.92 | 3.93 | 1.15 | 16.33 | 6.90 | 1.70 |
| WB/Xs079 | Purple | 68.58 | 48.08 | 28.67 | 37.25 | 23.67 | 28.17 | 8.25 | 9.33 | 4.21 | 1.35 | 12.25 | 6.82 | 1.45 |
| WB/Xs080 | Green | 70.92 | 55.83 | 27.42 | 36.86 | 21.44 | 24.92 | 9.25 | 9.83 | 3.53 | 1.05 | 12.50 | 6.07 | 1.08 |
| WB/Xs081 | Purple | 71.08 | 54.92 | 34.08 | 42.03 | 23.58 | 30.75 | 9.08 | 7.25 | 3.48 | 1.55 | 16.50 | 6.93 | 1.80 |
| WB/Xs082 | Purple | 76.92 | 59.42 | 36.58 | 39.89 | 22.08 | 32.58 | 8.25 | 8.17 | 3.61 | 0.98 | 13.17 | 5.95 | 1.23 |
| GQ/Xs083 | Green | 83.42 | 63.58 | 35.92 | 42.42 | 24.00 | 30.33 | 12.92 | 9.67 | 3.48 | 2.00 | 9.25 | 4.76 | 1.05 |
| GQ/Xs084 | Purple | 69.33 | 58.83 | 30.33 | 40.39 | 23.42 | 27.92 | 10.67 | 8.08 | 3.54 | 1.30 | 13.50 | 5.33 | 1.38 |
| GQ/Xs085 | Green | 79.67 | 60.25 | 32.67 | 38.50 | 23.28 | 28.83 | 12.58 | 10.42 | 3.48 | 1.50 | 16.92 | 5.78 | 0.88 |
| GQ/Xs086 | Purple | 68.92 | 54.92 | 28.83 | 40.97 | 23.75 | 32.50 | 9.75 | 10.17 | 4.05 | 1.10 | 15.25 | 6.30 | 1.35 |
| GQ/Xs087 | Purple | 82.50 | 67.83 | 40.50 | 42.39 | 23.75 | 34.00 | 9.08 | 8.42 | 3.94 | 1.15 | 13.83 | 6.74 | 1.28 |
| **S4 Table Continued** | | | | | | | | | | | | | | |
| GQ/Xs088 | Green | 79.92 | 54.33 | 29.75 | 36.42 | 22.67 | 31.92 | 9.75 | 8.25 | 2.73 | 1.03 | 11.08 | 4.89 | 0.95 |
| GQ/Xs089 | Purple | 68.34 | 58.75 | 30.33 | 38.22 | 21.67 | 32.08 | 10.17 | 8.58 | 3.73 | 1.40 | 13.50 | 6.67 | 1.25 |
| GQ/Xs090 | Green | 57.67 | 52.17 | 31.50 | 36.06 | 19.19 | 23.17 | 9.75 | 10.75 | 3.77 | 1.23 | 10.83 | 4.90 | 0.68 |
| GQ/Xs091 | Purple | 65.00 | 55.75 | 31.67 | 44.97 | 25.22 | 31.67 | 7.67 | 7.08 | 3.55 | 1.80 | 16.67 | 7.73 | 1.70 |
| GQ/Xs092 | Purple | 65.00 | 51.83 | 30.25 | 40.31 | 23.50 | 32.92 | 8.58 | 7.42 | 3.48 | 1.35 | 14.33 | 5.98 | 1.40 |
| GQ/Xs093 | Purple | 68.67 | 60.08 | 33.83 | 38.61 | 22.86 | 27.83 | 9.17 | 9.08 | 3.49 | 1.15 | 13.83 | 6.39 | 1.38 |
| GQ/Xs094 | Green | 71.92 | 54.58 | 29.58 | 39.42 | 22.44 | 27.75 | 12.67 | 11.17 | 3.41 | 1.73 | 10.50 | 5.66 | 1.23 |
| GD/Xs095 | Purple | 69.83 | 57.25 | 30.58 | 41.36 | 23.69 | 33.08 | 8.58 | 8.50 | 3.67 | 1.65 | 12.42 | 5.64 | 1.48 |
| GD/Xs096 | Purple | 67.56 | 58.75 | 28.25 | 35.06 | 20.08 | 28.83 | 9.00 | 7.67 | 3.61 | 1.23 | 13.33 | 6.17 | 1.15 |
| GD/Xs097 | Green | 75.58 | 58.25 | 31.67 | 43.00 | 24.83 | 30.08 | 7.75 | 11.08 | 3.98 | 1.10 | 8.75 | 5.28 | 0.75 |
| GD/Xs098 | Green | 61.08 | 49.25 | 28.67 | 32.58 | 18.67 | 22.50 | 6.83 | 8.50 | 2.83 | 1.03 | 8.33 | 3.75 | 0.75 |
| GD/Xs099 | Purple | 70.92 | 59.83 | 34.50 | 43.83 | 22.92 | 33.42 | 8.00 | 9.42 | 3.88 | 1.13 | 14.83 | 7.08 | 1.85 |
| GD/Xs100 | Green | 63.50 | 49.25 | 25.50 | 31.22 | 18.56 | 21.42 | 13.92 | 9.50 | 3.33 | 1.38 | 10.58 | 5.39 | 1.05 |
| **Grand mean** | | **71.49** | **55.22** | **31.25** | **37.25** | **22.02** | **28.83** | **10.05** | **9.05** | **3.26** | **1.28** | **11.94** | **5.70** | **1.18** |

PH= plant height, PL= petiole length, PSL=petiole sheath length, LL=lamina length, LW=lamina width, CAGS = circumference of above ground pseudo-stem, NC= number of cormels per plant, CL=cormel length, CD=cormel diameter, CFW=cormel fresh weight per plant, CrL= corm length, CrD=corm diameter, CrFW= corm fresh weight per plant.
