# Supplementary material for: Genetic diversity of Ethiopian cocoyam (Xanthosoma sagittifolium (L.) Schott) accessions as revealed by morphological traits and SSR markers
Source: PLoS One. 2021 Jan 7;16(1):e0245120. doi: 10.1371/journal.pone.0245120 (PMC7790241; doi:10.1371/journal.pone.0245120)
Supplement: Attachment — Submitted filename: PONE-D-20-24312_R1.pdf [file pone.0245120.s006.pdf]

PLOS ONE

Genetic diversity of Ethiopian cocoyam (Xanthosoma sagittifolium (L.) Schott) accessions as revealed by morphological traits and SSR markers

--Manuscript Draft--

**Manuscript Number:**	PONE-D-20-24312R1
**Article Type:**	Research Article
**Full Title:**	Genetic diversity of Ethiopian cocoyam (Xanthosoma sagittifolium (L.) Schott) accessions as revealed by morphological traits and SSR markers
**Short Title:**	Genetic diversity of Ethiopian cocoyam (Xanthosoma sagittifolium (L.) Schott)
**Corresponding Author:**	Eyasu Wada<br>Wolaita Sodo University<br>Wolaita Sodo, ETHIOPIA
**Keywords:**	Cocoyam, Genetic diversity, morphological traits, SSR markers
**Abstract:**	Cocoyam (Xanthosoma sagittifolium (L.) Schott) originated and first domesticated in tropical America. It is exotic to Ethiopia, but widely cultivated for its edible cormels and leaves. There is a dearth of information on the genetic diversity of Ethiopian cocoyam. In order to evaluate and select cocoyam germplsam for breeding and conservation, genetic diversity of 100 Ethiopian cocoyam accessions (65 green- and 35 purple-cocoyam) were analyzed using 29 morphological traits (16 qualitative and 13 quantitative) and 12 SSR loci. Two classes of qualitative traits were observed. ANOVA revealed significant variation in 11 (84.6%) of the 13 studied quantitative traits. The SSR marker analysis showed high genetic diversity. A total of 36 alleles were detected with range 2 to 5 alleles (an average of 3.273 alleles per locus). The average observed heterozygosity (Ho) and expected heterozygosity (He) values across populations were, 0. 503 and 0.443, respectively. The analysis of molecular variance showed that the variation among population explained 14%, while the variation from among individuals within populations and within individuals explained 18% and 68% of the total variation, respectively. Cluster analysis grouped the accessions irrespective of the collection sites. Dendrogram based on Nei's standard genetic distance grouped the green cocoyam accessions together while the purple cocoyam accessions occupied separate position within the dendrogram. Significant variation in quantitative traits and high level of genetic diversity revealed by SSR markers can fairly indicate that genetically diverse cocoyam accessions probably with multiple lineage might have been introduced multiple times, through multiple routes and probably by multiple agents. High genetic diversity should be considered as a good opportunity for breeding and conservation of cocoyam accessions. Further taxonomic and phylogenetic study is recommended to clarify the taxonomic group of Ethioan cocoyam accesions and to tress their evolutionary relationship with Xanthosoma species elsewhere.
**Order of Authors:**	Eyasu Wada
	Tileye Feyissa
	Kassahun Tesfaye
	Zemede Asfaw
	Daniel Potter
**Response to Reviewers:**	Response to Reviewers<br>First we would like to thank the reviewers for taking their time to give us these constructive comments. We have replied to the comments as follows:<br>•Response to general comments<br>o Our manuscript have been carefully checked and revised as needed according to the PLOS ONE's style requirements.<br>oThe financial disclosure statement is changed and the cover letter is updated<br>oFigure files are resubmitted carefully checking PLOS ONE requirements of figure files<br>oORCID included<br>oCaptions for supporting information files are included at the end of the manuscript and

Powered by Editorial Manager® and ProduXion Manager® from Aries Systems Corporation

in text citations are updated
Reply to reviewer's comment
Reviewer #1
•Clarify objective and elaborate hypothesis.
oObjective is clarified and hypothesis is elaborated
•Provide passport data?
oProvided as supplementary information (S1 Table. Passport data)
•Provide Map?
oProvided
•Improve Quality of dendograms and figures?
oImproved
•A reader may be confused about our study materials and feel that Colocasia might have been included because of a sentence in the introduction explaining that in Ethiopia the two species are considered synonymous.
oWe have edited the sentence and clarified it to avoid any confusion
•Size and diameter of corm?
oThe size and diameter of Xanthosoma corm is 10 to 15 cm in diameter but here the average diameter of corm recorded from 3 replication for two years experiment were 4.29 to 7.73 cm and the overall mean was 5.70 cm. Such difference may be due to:
1)Cocoyam is a perennial plant, but for practical purposes, the corm and cormles were intentionally measured separately at the end of 12th months after plantation (annually). This annual harvest could be the reason for the size and diameter reduction. Actually, we encountered larger sized corm at some fields where cocoyam grown as perennial crop when we were collecting germplasms, but which was not at research field (where it was cultivated annually).
2)The soil type of the experimental site could be another reason.
•One or several Xanthosoma species?
oIn general, the division of the genus Xanthosoma into species has been difficult [1]. There is much confusion, discrepancies and uncertainties regarding the taxonomy of Xanthosoma at the species level. Various names have been used synonymously [2-3], the same plant being given more than one species name [4]. Thus, the name of Xanthosoma sagittifolium (L.) Schott has usually been given to the most cultivated members of this genus (Giacometti and [5-6]. In the course of this study, we refer to the accepted species status of X. sagittifolium in The Plant List [2] in the Araceae family [6], which does not tackle taxonomy in further detail.
oThere was no substantial evidence to say that there were different Xanthosoma species in Ethiopia although qualitative morphological traits and SSR markers clustered accession into two groups. We have a plan to do taxonomic study on Xanthosoma species in Ethiopia. Generally, the taxonomy of Xanthosoma needs to be reconstructed.
1.Saborio, F. (2007). Cocoyam (Xanthosoma sagittifolium (L.) Schott). In: Breeding of Neglected and Underutilized Crops, Spices and Herbs, pp. 172-189, (Ochatt, S. and Jain, S.M., eds). Science Publishers, USA.
2.The Plant List (http://www.theplantlist.org)
3.Lim, T.K. (2015). Edible Medicinal and Non-Medicinal Plants. Volume 9: Modified Stems, Roots, Bulbs. New York: Springer.
4.O'Hair, S.K. and Maynard, D.N. (2003). Edible aroids. In: Encyclopedia of Food Science and Nutrition, PP. 5970-5973, (Trugo, L.C. Finglas, P.M. Belton, P. Ottaway, P.B., Bressani, R., et al., eds). Academic press, USA.
5.Giacometti, D.C. and Leon, J. (1994). Tannia, yautia (Xanthosoma sagittifolium). In: Neglected Crops 1492 from Different Perspectives, pp. 255–258, (Hernandez-Bermejo, L., ed). Food and Agricultural Organization of United the United Nations, Rome, Italy.
6.Govaerts, R., Frodin, D.G. and Bogner, J. (2002). World checklist and bibliography of Araceae (and Aroraceae). Royal Botanic Gardens, UK.
Reviewer #2
•Design and Replication?
oSimilar sized cormel germplasms were sampled from five Zones. Then, characterized using morphological traits by planting in a common research garden in 10x10 simple lattice square design for two years. This is now clarified in the methodology.
oThe genotype by environmental interaction (GXE) evaluation is the next plan to be done. Therefore, multilocational trials is to be conducted in different agroecology to assess the magnitude of genotype x environment (GXE) interaction and to identify stable genotypes and evaluate their performance, selecting some genotypes from the clustered accessions to evaluated yield and yield related traits.

Reviewer #3
Data availability
oAll data made to be available either as part of manuscript in table or as supplementary materials. This is also preferred comment from other two reviewers
Manuscript presentation
oSome improvements were made on the presentation of the paper as needed
Objective and Hypothesis
oObjective is clarified and hypothesis is elaborated
Sample collection zones
oThe accessions that were collected from the same zone were considered as one population for the genetic diversity studies in assumption that cormel germplasms were more likely shared within zones than among zones. The same language is spoken within the zones and there are frequent local markets within zones where local people buy and sell commodities. This is added in the methods and material section for clarity
Results
3.1 Start the result section with clear finding
oEdited to meet the reviver's comment
3.2 Please don't start a sentences with a Table
oThe comment is accepted and corrected
I would combine Table 2 and Table 3, emphasis to statistical significant character
oThe comment accepted and the two tabled combined. Emphasis was given to statistical significant character, where statistical comparison was made at p= 0.05, 0.01 and 0.001
3.3 Please mention the link to Table 4 early in the text
oThe link to table 4 is mentioned early in the text
Please check data and recalculate data for Ho =1
oData checked and recalculated for Ho =1 for two loci (mXsCIR22 and mXsCIR27) (Table 3) and six SSR markers presented Ho values higher than He values. SSR markers, which we have used were initially identified by Cathebras et al. [1], gave variable degrees of heterozygosity, observed at levels ranging from 0.00 to 0.97. This excess in heterozygotes might be due to high rates of asexual reproduction in cocoyam due to vegetative propagation. The inflorescence of cocoyam is protogynous [2].
1.Catherbras C. Traore C. Malapa R. Risterucci A. Chair H. characterization of microsatellites in Xanthosoma sagittifolium (Araceae) and cross-amplification in related species. Appl Plant Sci. 2014; 2(6); 2(6):129-134 http://doi.org/10.3732/apps.1400027.
2.Manner HI. Farm and forestry production and marketing profile for Tannia (Xanthosoma spp.). In: Elevitch CR, editor. Specialty Crops for Pacific Island Agroforestry. Permanent Agriculture Resources, Hawaii, USA; 2011. pp. 1-16.
How many samples from each population
oTwelve to twenty eight accessions were sample from each zone. We clarified this now in the methodology section and the accessions passport data is presented as supplementary information (S1 Table 1) according the reviewer #1 and Reviewer #2 comments
3.4 Don't repeat the values in the Table, extract the most important findings
oComment accepted accordingly
3.5 is not worth a separate chapter. Please combine with the previous
oCombined and the main finding is interpreted at the discussion section
3.6 Is this clustering of accessions separate from the morphological analysis?
oNo, clusters were done by using both morphological and SSR markers data. The results were presented by Fig. 2 and Fig. 3 to see how one goes with the other. It was seen that the second larger cluster (C-III) was contained 27 (27%) of the total accessions. Of the 27 accessions grouped in this cluster, 25 (92.3%) were purple-cocoyam accessions (Fig 2). This result was obtained by using data of morphological genetic traits. Molecular data was also grouped almost all purple-cocoyam accessions in one cluster and the green ones in another cluster (Fig.2), supporting the morphological data. These results were presented in the result section and possible reasons for this were explained in the discussion section.
In M&M it was not explained why clustering seems to be important
oChecked and explained
How clustering was conducted
oThe following sentences are in the M&M which explain how clustering of accession were conducted.
•The quantitative morphological traits were subjected to a cluster analysis using

	Minitab17.1, which was conducted by employing average linkage clustering strategy of the observation. Variables were standardized to a common scale by subtracting the means and dividing by the standard deviation. The number of clusters was determined by following the steps recommended by Minitab 17.1. The dendrogram showing the Euclidean distance between clusters was constructed by plotting the results of cluster analysis using the same program.<br>•The genetic clustering based on SSR data was assessed by performing using neighbour joining algorithm implemented in the computer program PHYLIP version 3.6 using Nei's genetic distance based on the frequencies generated by using MSA version 4.05.<br>Discussion<br>is too descriptive, several values that were mentioned in the result section were mentioned again, the discussion must be shortened, taxonomic conclusion need to be drawn<br>oThese comments were considered carefully and corrected accordingly.
Additional Information:	
Question	Response
**Financial Disclosure**<br><br>Enter a financial disclosure statement that describes the sources of funding for the work included in this submission. Review the submission guidelines for detailed requirements. View published research articles from *PLOS ONE* for specific examples.<br><br>This statement is required for submission and **will appear in the published article** if the submission is accepted. Please make sure it is accurate.<br><br>**Unfunded studies**<br>Enter: *The author(s) received no specific funding for this work.*<br><br>**Funded studies**<br>Enter a statement with the following details:<br>• Initials of the authors who received each award<br>• Grant numbers awarded to each author<br>• The full name of each funder<br>• URL of each funder website<br>• Did the sponsors or funders play any role in the study design, data collection and analysis, decision to publish, or preparation of the manuscript?<br>• **NO** - Include this sentence at the end of your statement: *The funders had no role in study design, data collection and analysis, decision to publish, or preparation of the manuscript.*<br>• **YES** - Specify the role(s) played.	would also like to disclose that<br>this study was supported by the DAAD in country/in region scholarship 2015 (Personal ref. no.:<br>91602027), Wolaita Sodo University provided vehicle for collection of accessions, the field<br>experiment was conducted at Areka Agricultural Research Center, SSR laboratory analysis was<br>conducted in Department of Plant Sciences of University of California-Davis and SSR fragment<br>analysis was done at the USDA laboratory, California.

* typeset	
**Competing Interests**<br><br>Use the instructions below to enter a competing interest statement for this submission. On behalf of all authors, disclose any competing interests that could be perceived to bias this work—acknowledging all financial support and any other relevant financial or non-financial competing interests.<br><br>This statement **will appear in the published article** if the submission is accepted. Please make sure it is accurate. View published research articles from *PLOS ONE* for specific examples.<br><br>**NO authors have competing interests**<br><br>Enter: *The authors have declared that no competing interests exist.*<br><br>**Authors with competing interests**<br><br>Enter competing interest details beginning with this statement:<br><br>*I have read the journal's policy and the authors of this manuscript have the following competing interests: [insert competing interests here]*<br><br>* typeset	The authors have declared that no competing interests exist
**Ethics Statement**<br><br>Enter an ethics statement for this submission. This statement is required if the study involved:<br><br>• Human participants<br>• Human specimens or tissue<br>• Vertebrate animals or cephalopods<br>• Vertebrate embryos or tissues<br>• Field research<br><br>Write "N/A" if the submission does not require an ethics statement.	N/A

General guidance is provided below. Consult the submission guidelines for detailed instructions. **Make sure that all information entered here is included in the Methods section of the manuscript.**<br><br>**Format for specific study types**<br><br>**Human Subject Research (involving human participants and/or tissue)**<br>• Give the name of the institutional review board or ethics committee that approved the study<br>• Include the approval number and/or a statement indicating approval of this research<br>• Indicate the form of consent obtained (written/oral) or the reason that consent was not obtained (e.g. the data were analyzed anonymously)<br><br>**Animal Research (involving vertebrate animals, embryos or tissues)**<br>• Provide the name of the Institutional Animal Care and Use Committee (IACUC) or other relevant ethics board that reviewed the study protocol, and indicate whether they approved this research or granted a formal waiver of ethical approval<br>• Include an approval number if one was obtained<br>• If the study involved *non-human primates*, add *additional details* about animal welfare and steps taken to ameliorate suffering<br>• If anesthesia, euthanasia, or any kind of animal sacrifice is part of the study, include briefly which substances and/or methods were applied<br><br>**Field Research**<br><br>Include the following details if this study involves the collection of plant, animal, or other materials from a natural setting:<br>• Field permit number<br>• Name of the institution or relevant body that granted permission	
**Data Availability**<br><br>Authors are required to make all data underlying the findings described fully	No - some restrictions will apply

Powered by Editorial Manager® and ProduXion Manager® from Aries Systems Corporation

available, without restriction, and from the time of publication. PLOS allows rare exceptions to address legal and ethical concerns. See the PLOS Data Policy and FAQ for detailed information.<br><br>A Data Availability Statement describing where the data can be found is required at submission. Your answers to this question constitute the Data Availability Statement and **will be published in the article**, if accepted.<br><br>**Important:** Stating 'data available on request from the author' is not sufficient. If your data are only available upon request, select 'No' for the first question and explain your exceptional situation in the text box.<br><br>Do the authors confirm that all data underlying the findings described in their manuscript are fully available without restriction?	
**Describe where the data may be found in full sentences. If you are copying our sample text, replace any instances of XXX with the appropriate details.**<br><br>• If the data are **held or will be held in a public repository**, include URLs, accession numbers or DOIs. If this information will only be available after acceptance, indicate this by ticking the box below. For example: *All XXX files are available from the XXX database (accession number(s) XXX, XXX.*).<br>• If the data are all contained **within the manuscript and/or Supporting Information files**, enter the following: *All relevant data are within the manuscript and its Supporting Information files.*<br>• If neither of these applies but you are able to provide **details of access elsewhere**, with or without limitations, please do so. For example:<br><br>*Data cannot be shared publicly because of [XXX]. Data are available from the XXX Institutional Data Access / Ethics*	All relevant data are within the manuscript and its Supporting Information files

Committee (contact via XXX) for researchers who meet the criteria for access to confidential data.<br><br>*The data underlying the results presented in the study are available from (include the name of the third party and contact information or URL).*<br>• This text is appropriate if the data are owned by a third party and authors do not have permission to share the data.<br><br>* typeset	
Additional data availability information:	

Powered by Editorial Manager® and ProduXion Manager® from Aries Systems Corporation

Manuscript Click here to access/download;Manuscript;Manuscript.docx

Genetic diversity of Ethiopian cocoyam (*Xanthosoma sagittifolium* (L.) Schott) accessions as revealed by morphological traits and SSR markers

Eyasu Wada[1]*, Tileye Feyissa[2], Kassahun Tesfaye[2], Zemede Asfaw[2] and Daniel Potter[4]

[1]Department of Biology, College of Natural Sciences, Wolaita Sodo University, Ethiopia

[2]Department of MCMB, College of Natural Sciences, Addis Ababa University, Ethiopia

[3]Department of Plant Biology and Biodiversity Management, College of Natural Sciences, Addis Ababa University, Ethiopia

[4]Department of Plant Sciences, College of Agricultural and Environmental Sciences, University of California-Davis, United States of America

*Corresponding author's email: eyasuwada@gmail.com (EW)

Abstract

Cocoyam (*Xanthosoma sagittifolium* (L.) Schott) originated and first domesticated in tropical America. It is exotic to Ethiopia, but widely cultivated for its edible cormels and leaves. There is a dearth of information on the genetic diversity of Ethiopian cocoyam. In order to evaluate and select cocoyam germplsam for breeding and conservation, genetic diversity of 100 Ethiopian cocoyam accessions (65 green- and 35 purple- cocoyam) were analyzed using 29 morphological traits (16 qualitative and 13 quantitative) and 12 SSR loci. Two classes of qualitative traits were observed. ANOVA revealed significant variation in 11 (84.6%) of the 13 studied quantitative traits. The SSR marker analysis showed high genetic diversity. A total of 36 alleles were detected with range 2 to 5 alleles (an average of 3.273 alleles per locus). The average observed heterozygosity (Ho) and expected heterozygosity (He) values across populations were, 0. 503 and 0.443, respectively. The analysis of molecular variance showed that the variation among population explained 14%, while the variation from among individuals within populations and within individuals explained 18% and 68% of the total variation, respectively. Cluster analysis grouped the accessions irrespective of the collection sites. Dendrogram based on Nei's standard genetic distance grouped the green cocoyam accessions together while the purple cocoyam accessions occupied separate position within the dendrogram. Significant variation in quantitative traits and high level of genetic diversity revealed by SSR markers can fairly indicate that genetically diverse cocoyam accessions probably with multiple lineage might have been introduced multiple times, through multiple routes and probably by multiple agents. High genetic diversity should be considered as a good opportunity for breeding and conservation of cocoyam accessions. Further taxonomic and phylogenetic study is recommended to clarify the taxonomic group of Ethioan cocoyam accesions and to tress their evolutionary relationship with *Xanthosoma* species elsewhere.

1. Introduction

Cocoyam (Xanthosoma sagittifolium (L.) (Schott) is an herbaceous, monocotyledons crop in the Araceae family. Cocoyam originated and first domesticated in tropical America, where it was cultivated from very ancient times [1]. It is one of the oldest cultivated aroid in the world [2]. Nowadays, it is cultivated in many tropical countries for its edible cormels and leaves [3].

There is much confusion, discrepancies and uncertainties regarding the taxonomy of *Xanthosoma* at the species level [4]. Many names are associated with the cultivated species of the genus *Xanthosoma* [5-6] but the name *Xanthosoma sagittifolium (*L.) (Schott) has usually been given to all cultivated species of the genus [6] and several common names are also used to refer the cultivated species [5, 7-10]. In the course of this study, cocoyam is ~~used to~~ refere to *X. sagittifolium*~~-~~, which is accepted ~~species~~ name of cultivated species of the genus *Xanthosoma* in the Araceae family [11] and in The Plant List [12].

Cocoyam was introduced into Eastern Africa through Western Africa [13]. In Ethiopia, cocoyam is largely unknown or considered synonymously with other related aroid, *Colocasia esculenta* (L.) Schott), which is locally known as taro or "*Godere*", [14]. However, cocoyam can be distinguished morphologically from taro by the place where the petiole meets the leaf. In cocoyam, the petiole attachment is at the margin of the leaf between the lobes. In taro, the petiole attachment is peltate or around the middle of the leaf [5].

Cocoyam flowers rarely. When occurs, the flowers are monoecious within a compound inflorescence. The female flowers are at the base of the spadix, and the male flowers are above. Sterile flowers are located between the pistillated and staminated flowers. The inflorescence is protogynous. The stigma is normally receptive two to four days before pollen shed [15]. This makes the classical breeding difficult. Cocoyam rarely set sexual seeds and hence it is mainly propagated vegetatively from corm sets, headsets or cormels [16] but

treatments were successfully applied to induce a flowering with artificial pollination techniques. Hand pollination and production of seed and seedlings has become possible at experimental settings [17-18].

Cocoyam grows in a variety of substrates and habitats in full sun or deeply shaded areas under the canopy of natural forests. It tolerates drier, but not waterlogged soils [7]. It is a lowland plant but grows in highlands [15]. It is a perennial plant but most often cultivated as an annual crop for food value. Harvest occurs during the dry season, 9-12 months after plantation [9]. At the end of the growing season, leaves die and shoots wither completely [16, 19].

Many developing countries in the tropics depend on aroids in the tribes *Colocasieae* and *Caladieae*, i.e., taro (*C. esculenta*) and cocoyam (*X. sagittifolium*) as sources of carbohydrate [20]. Cocoyam has overtaken taro as the main edible aroid in many tropical areas [3]. It is an important crop in many parts of the tropical world, mainly for smallholder farmers, in playing a major role in the lives of many as a food security crop. Both its leaves and its starch-rich cormels are eaten after cooking [2-3].

In Ethiopia, cocoyam has spread widely and become an important part of the agriculture and food systems of the local people. There is considerable amount of cocoyam gene pool in farmers' fields [21-28] but did not receive thoughtful research effort as it is a neglected crop by research and development community. The germplasms in the farmers' field are the major hope for use, conservation and improvement of the crop. Thus, collecting accessions and assessing genetic diversity are very important to identify germplasms for efficient and effective usage, conservation and improvement.

Morphological traits are conventional tools to analyze the genetic diversity by measuring the variation among accessions [29]. It is an essential step for effective utilization of germplasms because it offers a useful approach for assessing the extent of diversity [30].

The genetic information provided by morphological traits is, however, often limited and expression of quantitative traits is subjected to strong environmental influence [31-32]. Assessing genetic diversity of plant species by using molecular markers has several advantages over morphological traits based characterization [33] although they cannot completely replace it. Complementary to morphological traits based characterization, the use of molecular markers has been importantly increasing [31]. This study employed genetic characterization using morphological descriptors and SSR molecular markers to assess the genetic diversity of Ethiopian cocoyam (*Xanthosoma sagittifolium* (L.) Schott) accessions to enhance effective usage, conservation and improvement of cocoyam.

2. Materials and methods

2.1 Plant materials

Similar sized cormel germplasms of 100 cocoyam accessions were sampled from 5 zones (Bench-Maji, Kefa, Dawuro, Wolaita and Gamo-Gofa) (Fig. 1). Twelve to twenty-eight accessions were sampled from each zone (S1 Table). The individual accessions were collected from at least 5 km apart unless they were clearly distinguished by leaf and petiole color difference (65 green- cocoyam and 35 purple-cocoyam). The accessions that were collected from the same zone were considered as one population for the genetic diversity studies in assumption that cormel germplasms were more likely shared within zones than among zones. The same language is spoken within the zones and there are frequent local markets within zones, where local people buy and sell commodities. The accessions are kept as living material at the Areka Agricultural Research Center.

Fig. 1 Map representing the collection Zones of the cocoyam accessions. Twelve to twenty-eight accessions were sampled from each Zone (S1 Table). The individual accessions were collected from at least 5 km apart unless they were clearly distinguished by leaf and petiole color difference.

2.2 Experimental design and crop management

The cormel germplasms (100) were planted in a common research garden at Areka Agricultural Research Center, 303 km southwest of Addis Ababa, Ethiopia in a 10*10 simple lattice square design in well-drained, loose soil on flat ground in 2016 cropping season. Five cormels of an accession were planted in a single row plot of 3 m, spaced 0.6 m between plants and 0.75 m between rows. Weeding was conducted as required to keep plots weed free. Similar sized cormels were harvested at the end of the 12th month after the date plantation and the experiment was repeated in 2017 cropping season using the same experimental design and taking similar crop management practices.

2.3 Morphological traits and data collection

Twenty-nine morphological traits (S2 Table) were selected from International Board for Plant Genetic Resources Descriptors for *Xanthosoma* [34]. The qualitative data were scored for qualitative traits such as plant growth habit, petiole attachment, petiole color (upper 2/3rd), petiole color (lower 1/3rd), color of the edge of petiole, lamina orientation, leaf margin color, leaf shape, color of upper leaf surface, color of lower leaf surface, position of cormel apex, shape of cormels, color of cormel apex and fresh cormel color at the end of the 7th month after the date of plantation, from the middle individual plant. The Munsell Plant Tissue Color Chart [35] was used to discriminate colors. The quantitative data were recorded from the middle three plants of each plot, leaving the two plants that grown as boarder plants. The aboveground quantitative traits such as overall plant height (cm), petiole length (cm), petiole sheath length (cm), circumference of the pseudo-stem (cm), lamina length (cm) and lamina width (cm) were measured at the end of the 7th month after the date of plantation. The underground quantitative traits such as the number of cormels/plant, cormel length (cm), cormel diameter (cm), cormel fresh weight/plant (kg), corm length (cm), corm diameter (cm)

and corm fresh weight/plant (kg) were recorded at the end of 11th months after date of plantation.

2.4 DNA extraction

Young leaf samples were collected by using silica gel (Bio lab). Total genomic DNA was extracted using DNeasy plant min kit (QIAGEN) according to the manufacture's instruction. The DNA was checked on a 2% agarose gel. The quantity and quality of DNA were assessed by using Nanodrop-2000.

2.5 SSR primers and PCR amplification

Seventeen microsatellite primer pairs identified by Cathebras et al. [36] for *X. sagittifolium* were tested with 5 randomly selected samples. Twelve SSR primer pairs (S3 Table), which gave an intense band on an agarose gel, were selected for subsequent analysis. The forward primer of each of the 12 pairs was labeled either with 6-FAM (6-Carboxyfluorescein) or Hexachloro-fluorescein (HEX). The markers were paired into 6 PCR sets, each set containing 6-FAM labeled primer pair and HEX labeled primer pair. These labeled primers were used to test 15 samples in a pre-analysis.

PCR was performed in a total volume of 20 µL containing 2 µL of 10x PCR buffer (15 mM $MgCl_2$ included), 0.4 µL dNTP mix (10 mM each), 0.5 µL (16 pmol/µL) of each of the forward and reverse SSR primers, 2 µL genomic DNA (20 ng/µL), 0.2 µL Taq polymerase and 14.4 µL ddH_2O. Amplification was performed in ABI 2720 Thermocyler. After initial denaturation at 95 °C for 2 min, 30 cycles denaturation at 94 °C for 30 s, annealing at 52 °C for 45 s, extension at 72 °C for 1 min followed with a final extension of 72 °C for 10 min were used. After verification of PCR amplification through 1% agarose gel electrophoresis, capillary electrophoresis was run by mixing the aliquots of PCR product (1 µL/well) with formamide (12 µL/well) and GeneScan™ 400HD ROX™ Size Standard (0.4 µL/well) in a 96 plate. The contents were denatured using a Boekel dry bath, which was heated to 95 °C for 5

min. Then, the DNA was quenched at -20 °C for 5 min and electrophoresed on an ABI 3130XL Genetic Analyzer (Applied Biosytems). The number and sizes of electrophoresed DNA fragments were identified using GeneMapper version 4.0 [37].

2.6 Data analysis

Frequency distribution of qualitative traits and basic statistical parameters based on quantitative morphological traits were calculated using SPSS version 23 [38]. Analysis of variance (ANOVA) was computed by PROC GLM procedure of SAS 9.3 [39] because the relative efficiency of the lattice design compared with CRBD was low (less than 105%). The number of alleles (Na), the number of effective allele (Ne) [40], Shanon information index (I) [41], observed (Ho) and expected (He) heterozygosity within individual populations, were generated using the program POPGENE version 1.32 [42]. Analysis of molecular variance (AMOVA) was performed to evaluate the partitioning of molecular variation within and among populations using GenAlEx version 6.503 [43].

Clustering, which is an important clue for visual perception was constructed morphological as well as SSR marker data. The quantitative morphological traits were subjected to a cluster analysis using Minitab17.1 [44] by employing average linkage clustering strategy of the observation. Variables were standardized to a common scale by subtracting the means and dividing by the standard deviation. The number of clusters was determined by following the steps recommended by Minitab 17.1 [44]. The dendrogram showing the Euclidean distance between clusters was constructed by plotting the results of cluster analysis using the same program. The genetic clustering based on SSR data was assessed by performing using neighbor joining algorithm implemented in the computer program PHYLIP version 3.6 [45] using Nei's genetic distance [46] based on the frequencies generated by using MSA version 4.05 [47].

3.Results

3.1 Qualitative traits

Of 16 qualitative traits, 9 discriminated the cocoyam accessions into two groups, where 35 purple cocoyam accesions were grouped together and 65 green ccocoyam accessions ~~were~~ occupied another group.Two classes of petiole color (upper 2/3rd), petiole color (lower 1/3rd), color of edge of petiole sheath, lamina orientation, color of upper leaf surface, color of lower leaf surface, color of veins on lower leaf surface, position of color apex and shape of cormels existed in the accessions. Qualitative traits such as plant growth habit, petiole attachment, leaf shape, leaf margin color, color of veins on upper leaf surface, color of cormel apex and fresh cormel color did not discriminate the accessions included in this study (Table 1).

Table 1. Frequency distribution of 16 qualitative traits of cocoyam accessions.

No	Plant character	Character state	%*
1	Plant growth habit	Acaulescent	100
2	Petiole attachment	Non-peltate	100
3	Petiole color (upper 2/3rd)	Green	65
		Purple	35
4	Petiole color (lower 1/3rd)	Green streaked with purple	65
		Purple	35
5	Color of edge of petiole sheath	The same as the rest of petiole and sheath	35
		Purple	65
6	Lamina orientation	One plane - apex down (Droopy)	35
		3-dimentional (cup-shaped)	65
7	Leaf shape	Sagitate basal lobes	100
8	Leaf margin color	Purple edge	100
9	Color of upper leaf surface	Medium green	65
		Dark green	35
10	Color of lower leaf surface	Light green	65
		Purplish green	35
11	Color of veins on upper leaf surface	Lighter green than lamina	100
12	Color of veins on lower leaf surface	Same as color as lamina	65
		Purple	35
13	Position of color apex	Underground	65
		Above ground and under ground	35
14	Shape of cormels	Globose	65
		Ovate	35
15	Color of cormel apex	Red	100
16	Internal color of cormels	White streaked with purple	100

*100% indicates the character common to both green leaf/petiole-cocoyam and purple leaf petiole-cocoyam accesions, 65% character specific to green- cocoyam accessions and 35% character specific to purple- cocoyam accessions.

3.2 Descriptive statistical parameters and variance

Descriptive statistics showed that the mean values above ground quantitative traits such of plant height, petiole length (cm) petiole sheath length (cm), lamina length (cm) lamina width (cm) and circumference of pseudo-stem (cm) were 71.49, 55.22, 31.24, 37.77, 22.02 and 28.83, respectively. The mean values of underground quantitative traits such as the number of cormels/plant, cormel length (cm), cormel diameter (cm), cormel fresh weight/plant (kg), corm length (cm), corm diameter (cm) and corm fresh weight/plant (kg), respectively, were 10.05, 9.05, 3.26, 1.28, 11.94, 5.70 and 1.18. Maximum SD (5.63) corresponded to plant height and minimum SD (0.31) corresponded to cormel fresh weight/plant (kg). The coefficient of variation (CV %) varied from 7.65% for lamina width to 29.10% for the corm fresh weight/plant. Analysis of variance (ANOVA) revealed significant variation in 11(84.6%) of the 13 studied quantitative traits (Table 2).

Table 2. Basic statistics and summary of mean squares of quantitative traits

Quantitative traits	Mean	SD	Minimum	Maximum	CV%	Mean squares		
						Rep (df =1)	Accessions (df =99)	Error (df =81)
PH	71.49	5.63	57.58	84.83	7.87	100.30	82.46*	54.46
PL	55.22	4.82	42.75	67.83	8.72	294.64	49.57*	31.66
PSL	31.24	3.46	25.50	43.92	11.08	333.62	28.16	21.48
LL	37.77	3.20	29.69	45.25	8.48	20.09	16.73***	8.10
LW	22.02	1.68	17.28	25.47	7.65	0.74	4.96***	2.41
CPS	28.83	2.78	21.42	34.42	9.66	128.27	14.02***	5.72
CN	10.05	1.81	5.92	15.33	17.98	5.01	7.40**	3.91
CL	9.05	1.09	6.58	11.83	12.00	0.02	2.17	2.30
CD	3.26	0.46	2.25	4.21	13.97	1.12	0.29**	0.17
CFW	1.28	0.31	0.68	2.42	23.79	1.71	0.23**	0.13
CrL	11.94	2.11	3.49	16.92	17.71	102.84	7.65**	4.38
CrD	5.70	0.77	3.49	7.73	13.52	2.95	1.06***	0.45
CrFW	1.18	0.34	0.53	2.30	29.10	1.98	0.24***	0.12

PH- plant height (cm), PL-petiole length (cm), PSL-petiole heath length (cm), LL-lamina length (cm), LW-lamina width (cm), CPS-circumference of pseudo-stem (cm), CN- cormel Number per plant, CL-cormel length (cm), Cormel Diameter (cm), CFW-cormel fresh weight per plant (kg), CrL-corm length (cm), CrD-corm diameter (cm), CrFW-corm fresh weight/plant(kg), df - degree of freedom, *****, ****** and ******* significant at p= 0.05, 0.01 and 0.001, respectively.

3.3 Genetic diversity of cocoyam as revealed by SSR markers

Among a total of 12 SSR loci, 11 (91.7%) showed polymorphism. A total of 36 alleles, ranged from 2 to 5 per locus with an average of 3.273, were detected (Table 3). The Ne per locus ranged from 1.412 (locus mXsCIR07) to 3.759 (locus mXsCIR22) with mean Ne of 2.516. The observed heterozygosity (Ho) ranged from 0.000 (loci mXsCIR19, mXsCIR16 and mXsCIR24) to 1.000 (loci mXsCIR22 and mXsCIR27) with mean Ho of 0.508. The expected heterozygosity (He) ranged from 0.294 (locus mXsCIR07) to 0.733 (locus mXsCIR22) with mean He of 0.566.

Table 3. Genetic diversity parameters in 100 cocoyam accessions

Locus name	Na	Ne	I	Ho	He
mXsCIR05	4	3.499	1.304	0.800	0.720
mXsCIR07	2	1.412	0.468	0.355	0.294
mXsCIR19	2	1.851	0.652	0.000	0.462
mXsCIR10	4	2.810	1.087	0.905	0.648
mXsCIR21	4	2.498	1.098	0.372	0.603
mXsCIR22	4	3.759	1.354	1.000	0.738
mXsCIR11	4	2.158	0.969	0.650	0.539
mXsCIR24	2	1.821	0.643	0.000	0.453
mXsCIR12	3	2.368	0.972	0.505	0.581
mXsCIR27	5	3.689	1.364	1.000	0.733
mXsCIR16	2	1.807	0.639	0.000	0.449
Mean	3.273	2.516	0.959	0.508	0.566
SD	1.103	0.823	0.318	0.395	0.142

Na - number of different alleles, Ne - effective number of alleles, I - Shannon information index, Ho- observed heterozygosity and He - expected heterozygosity.

3.4 Genetic diversity within populations

The observed number of alleles (Na) varied from 1.727 (Bench-Maji, Kefa) to 3.091 (Gamo-Gofa) and the effective number of alleles (Ne) varied from 1.560 (Kefa) to 2.660 (Gamo-Gofa), with mean Na and Ne across populations were 2.455 and 2.091, respectively. The expected heterozygosity (He) values ranged from 0.267 (Bench-Maji) to 0.608 (Gamo-Gofa), mean He was 0.443. Shannon diversity index reflected similar trend of He (Table 4). The partitioning of molecular variance (AMOVA) showed that the highest genetic variability

(68%) existed within individuals and the lowest variability (14%) existed among populations (Table 5).

Table 4. Genetic diversity within populations.

Population	N	Na	Ne	Ho	He	I
Bench-Maji	14	1.727	1.631	0.446	0.267	0.391
Kefa	12	1.727	1.560	0.515	0.289	0.399
Dawuro	28	2.818	2.093	0.492	0.476	0.787
Wolaita	28	2.909	2.513	0.520	0.574	0.935
Gamo-Gofa	18	3.091	2.660	0.540	0.608	0.990
Average across populations	20	2.455	2.091	0.503	0.443	0.700
At species level	100	3.273	2.516	0.508	0.566	0.959

N - number of accessions; Na - number of different alleles; Ne - effective number of alleles; Ho- observed heterozygosity; He - expected heterozygosity; I - Shannon information index.

Table 5. Summary of AMOVA for five cocoyam populations based on SSR markers

Source of variation	df	Sum of squares	Estimated variance	% of variation	Fst	P value
Among Pops	4	94.046	0.510	14	0.143	0.001
Among Indivs	95	349.994	0.637	18		
Within Indivs	100	241.000	2.410	68		
Total	199	685.040	3.558			

3.5 Clustering of accessions

The cluster analysis based on the mean values of 13 quantitative morphological traits grouped the 100 cocoyam accessions into 4 clusters (Fig. 2). The clustering means of accessions based on 13 quantitative traits is presented in Table 6. Cluster four (C-IV) was the largest cluster composed of 41 (41%) of accessions and the least compacted ~~cluster than~~ ~~all other~~ clusters, having the largest within cluster sum of squares. Accessions in this cluster were characterized by the highest mean value for number of cormels/plant. Cluster three (C-III) was the second ~~larger~~ cluster contain~~ed~~ 27 (27%) of the total accessions. Of the 27 accessions grouped in this cluster, 25 (92.3%) were purple- cocoyam accessions. Cluster two (C-II) included the smallest number of accessions (11%) ~~than~~ all other clusters and it is a more compact cluster, which has the smallest within cluster sum of squares. Accessions in this cluster were characterized by the highest mean values for plant height, petiole length, lamina length and

width, circumference of pseudo-stem and corm length. Cluster one (C-I) contained 21 (21%) accessions, which were characterized by the lowest mean values for most of the traits studied. The cluster analysis showed that most of the accessions collected from different zones (populations) were clustered together. Individual accessions clustering based on SSR data is presented in the neighbor joining (NJ) tree based on Nei's genetic distance. The clustering fully separated green- cocoyam accessions from the purple- cocoyam accessions (Fig. 3).

Table 6. Cluster means of 13 quantitative traits of cocoyam.

**Quantitative traits**	**Means values of cluster**			
	**C-I**	**C-II**	**C-III**	**C-IV**
PH	67.26	80.00	67.60	73.93
PL	50.16	63.16	55.15	55.51
PSL	38.44	37.86	30.80	31.21
LL	34.50	41.13	39.71	37.28
LW	20.33	23.89	22.63	22.14
CPS	26.00	31.69	30.42	28.48
CN	9.19	10.32	9.28	10.93
CL	9.03	9.61	8.60	9.21
CD	2.91	3.59	3.68	3.08
CFW	1.02	1.43	1.31	1.35
CrL	10.27	12.30	14.08	11.28
CrD	5.00	6.19	6.31	5.55
CrFW	0.92	1.29	1.51	1.07
Within cluster sum of squares	167.63	117.39	192.94	304.46

PH- plant height (cm), PL-petiole length (cm), PSL-petiole heath length (cm), LL-lamina length (cm), LW-lamina width (cm), CPS-circumference of pseudo-stem (cm), CN- cormel Number per plant, CL-cormel length (cm), Cormel Diameter (cm), CFW-cormel fresh weight per plant (kg), CrL-corm length (cm), CrD-corm diameter (cm) and CrFW-corm fresh weight/plant(kg).

Fig. 2 Cluster analysis showing the relationship among cocoyam accessions based on quantitative traits. The numbers stand for the serial numbers of the accession code. The accessions 1-14, 15-26, 27-54, 55-82 and 83-100 were from Bench-maji, Kefa, Dawuro, Wolaita and Gamo-Gofa zones, respectively.

Fig. 3 Neighbour Joinings tree showing the relationships among Ethiopian cocoyam accessions. The tree was generated based on Nei's genetic distance using 11 microsatellite markers. Green- and purple- represent green- and purple- cocoyam accessions. The accessions Xs001-Xs014, Xs015-Xs026, Xs027-Xs054, Xs055-Xs082 Wolaita and Xs083 -Xs100 were from Bench-maji, Kefa, Dawuro, Wolaita and Gamo-Gofa zones, respectively.

4. Discussion

This study employed the techniques of genetic characterization using morphological traits and SSR molecular markers, which provided insight into a broader context for the genetic diversity and clustering of cocoyam accessions from Ethiopia. The majority (84.6%) of the quantitative morphological traits showed significant variation among accessions. Similar to this finding other authors, who had assessed genetic diversity of cocoyam accession by using morphological traits also reported significance variation among the studied accessions [24, 48-49]. SSR markers revealed high genetic diversity across populations. Most of (91.7%) the SSR loci were polymorphic within cocoyam accessions. In related study, Doungous et al. [50] reported 88% to 97% polymorphic loci across 20 cocoyam accessions from Cameroon using retrotransposon molecular markers. The avergae He across populations (He = 0.443) was higher than that of *Ambrosina bassii* L. (Araceae, Ambrosineae) (He = 0.263) [51] and *Anthurium crenatum* (L.) Kunth (Araceae) (He = 0.167) [52], implying that our collections contained genetically diverse cocoyam accessions. The observed heterozygosity was very high (Ho = 1) for two loci (mXsCIR22 and mXsCIR27) (Table 3) and six SSR markers presented Ho values higher than He values. SSR markers identified by Cathebras et al. [36], to assess cocoyam genetic diversity, gave variable degrees of heterozygosity, observed at levels ranging from 0.000 to 0.970. This excess in heterozygotes might be due to high rates of asexual reproduction in cocoyam due to vegetative propagation. The inflorescence of cocoyam is protogynous[15].

The extent of genetic clustering is of great interest in a number of biological fields including evolution, conservation and breeding [53]. The qualititative morphological traits and SSR markers-based clustering analyses effectively separated the purple cocoyam accessions from green cocoyam accessions. Cluster analysis based on quantitative morphological data also grouped most of (92.3%) the purple cocoyam accessions in distinct

cluster (cluster III), suggesting the existence of genetic difference between green-and purple-cocoyam accessions. The color difference could be considered as important for easy identification of accessions. Most of the accessions collected from different zones (populations) were collected from different zones, were grouped regardless of the collection zones, indicating that there have been movement of germplasm among zones , possibly by farmer-to-farmer planting materials exchange. Previously, a similar study [26] showed that most of the cocoyam accessions from different districts and villages of Ethiopia were clustered together.

~~For the reason that~~ the exact date of cocoyam introduction into Ethiopia and who introduced it cannot be unequivocally ~~spelt out~~, high genetic diversity among cocoyam accession and a significant differentiation between the morphotypes (green- and purple cocoyam) can ~~fairly~~ indicate that geneticaly diverse cocoyam accessions probably with multiple lineage might have been introduced into the country multiple times, through multiple routes and probably by multiple agents. High genetic diversity should be considered as a good opportunity for breeding, conservation and improvement of cocoyam. Further taxonomic and phylogenetic study is recommended to clarify the taxonomic group of cocoyam landraces of Ethiopia and to ~~tress~~ their evolutionary relationship with *Xanthosoma* species elsewhere.

Acknowledgement

The authors appreciate Dr. Asfaw Kifle for the assistance during the field works and during morphologicla data collection.

References

1. Giacometti DC, Leon J. Tannia, yautia (*Xanthosoma sagittifolium*). In: Hernandez-Bermejo L, editor. Neglected Crops 1492 from Different Perspectives. Food and Agricultural Organization of United the United Nations, Rome, Italy; 1994. pp. 255–258.
2. Purseglove JW. Tropical crops. Monocotyledons. Longman, London, UK; 1972.

3. Ramawat KG, Merillon JM. Bulbous Plants Biotechnology. CRC press, Taylor and Francis group, USA; 2014.
4. Saborio F. Cocoyam (*Xanthosoma sagittifolium* (L.) Schott). In: Ochatt S and Jain SM editors. Breeding of Neglected and Underutilized Crops, Spices and Herbs, pp. Science Publishers, USA; 2007. pp.172-189.
5. Mayo SJ, Bogner J, Boyce PC. The Genera of Araceae. European Union by Continental Printing, Belgium; 1997.
6. Quero-Garcia J, Ivancic Q, Lebot V. Taro and Cocoyam. In: Bradishaw JE, editor. Hand Book of Plant Breeding: Root and Tuber Crops. Springer Science and Business Media, London, UK; 2010. pp. 149–172.
7. Morton JF. Cocoyams (*Xanthosoma caracu*, *X. atrovirens* and *X. nigrum*), ancient root and leaf vegetables, gaining in economic importance. Flori Stat Hortic Socie. 1972; 85: 85–94.
8. Bown D. Aroids: Plants of the Arum Family, Second ed. Timber Press, Portland Oregon, USA; 2000.
9. Raemaekers RH. Crop Production in Tropical Africa. Ministry of Foreign Affairs, External Trade and International Co-operation, Directorate General for International Co-operation, Brussels, Belgium; 2001.
10. Lebot V. The Tropical Root and Tuber Crops: Cassava, Sweet Potato, Yams and Aroids. Crop Production Science in Horticulture Series No 17, CABI, UK; 2009.
11. Govaerts R, Frodin DG, Bogner J. World checklist and bibliography of Araceae (and Aroraceae). Royal Botanic Gardens, UK; 2002.
12. The Plant List (http://www.theplantlist.org).
13. Maundu P, Achigan-Dako E, Morimoto Y. Biodiversity of African Vegetables. In: Shackleton CM, Pasquini M, Drescher AW, editors). African Indigenous Vegetables in Urban Agriculture, Earthscan, UK; 2009. pp. 65 –105.
14. Simone A. Taro roots in North Omo. FPR Technical Pamphlet No. 2, Addis Ababa, Ethiopia; 1992.
15. Manner HI. Farm and forestry production and marketing profile for Tannia (*Xanthosoma* spp.). In: Elevitch CR, editor. Specialty Crops for Pacific Island Agroforestry. Permanent Agriculture Resources, Hawaii, USA; 2011. pp. 1-16.
16. Jackson GV. Major aroids. In: Dulloo ME. Thormann I. Jorge MA. Hanson J, editors. Crop Specific Regeneration Guidelines. CGIAR System-Wide Genetic Resource Programme, Rome, Italy; 2008. pp. 1-16.
17. Wilson JE. Promotion of flowering and production of seed in cocoyam (*Xanthosoma* and *Colocasia*). International Symposium on Tropical Root and Tuber Crops, IITA, Ibadan, Nigeria; 1979.
18. Goenaga R. Hepperly P. Flowering induction, pollen and seed viability and artificial hybridization of taniers (*Xanthosoma* spp.). J Agric Uni Puerto Rico. 1990; 74(3): 253–260.
19. Onwueme IC. Charles WB. Tropical Root and Tuber Crops: Production, Perspectives and Future Prospects. Agricultural Organization of United the United Nations, Rome, Italy; 1994.

20. Goenaga R. Singh U. Beinroth FH. Prasad H. Substore-Aroid: A model in the making. Agrobiotechnology transfer 1991. 14: 1-4.

21. Asfaw Z. Origin and evolution of rural home-garden in Ethiopia. In: Friis I, editor, Biodiversity Research in the Horn of Africa Region. University of Copenhagen, Denmark; 2001. pp. 273–286.

22. Asfaw Z. Home gardens in Ethiopia: Some observations and generalizations In: Watson JW. Eyzaguirre PB, editors. Home Gardens and *in situ* Conservation of Plant Genetic Resources in Farming Systems. Proceedings of the Second International Home-Gardens Workshop, Witzenhausen, Germany; 2001. pp.125–139.

23. Nebiyu A. Awas T. Exploration and collection of root and tuber crops in Southwestern Ethiopia: its implication for conservation and research, in: Proceeding of the 11th Conference of the Crop Science Society of Ethiopia, 26-28 April, 2004, Addis Ababa, Ethiopia; 2006.

24. Nebiyu A. Gardew W. Tofu A. Abebe W. Kifle A. Etissa E. Variety development for other root and tuber crops (taro, cassava and yam). In: Woldegiorgis G. Gebre E. Lemaga B, editors. Root and Tuber Crops: The Untapped Resources. EIAR, Addis Ababa, Ethiopia; 2008. pp. 303–316.

25. Fujimoto T. Taro (*Colocasia esculenta* (L.) Schott) cultivation in vertical wet-dry environments: Farmers' techniques and cultivar diversity in southwestern Ethiopia. Econ Bot. 2009; 63(2):152–166. http://doi.org/10.1007/s12231-009-9074-7

26. Fantaw F. Nebiyu A. Mulualem T. Genetic diversity of Tannia (*X. sagittifolium* (L.) Schott) genotypes using multivariate analysis at Jimma, South west Ethiopia. Int J Plant Breed Genet. 2014; 8(4):194–204. http://doi.org/10.3923/ijpbg.2014.194.204

27. Fantaw S. Nebiyu A. and Mulualem T. Estimate of genetic components of yield and yield related traits of tannia (*Xanthosoma sagittifolium* (L.) Schott) genotypes at Jimma, Southwest Ethiopia. Afr J Agric Res. 2014;10(1):23–30. http://doi.org/10.5897/AJAR2014.8997

28. Woldeyes F. Asfaw Z. Demisew S. Bernard R. Home-gardens of the Basketo People of southwestern Ethiopia: Sustainable agro-ecosystems characterizing a traditional landscape. Ethnobot Res Appl. 2016; 14:549–563. http://doi.org/10.17348/era.14.0.549-563

29. Sinha S. Kumaravadivel N. Understanding genetic diversity of sorghum using quantitative traits. Scientifica. 2016. http://doi.org/10.1155/2016/3075023

30. Koorneef M. Stam P. Changing paradigms in plant breeding. Plant Physiol. 2001; 125:156–159. http://doi.org/10.1104/pp.125.1.156

31. Karp A. Kresovich S. Bhat K.V. Ayad W.G. Hodgkin T. Molecular tools in plant genetic resources conservation: a guide to the technologies. International Plant Genetic Resources Institute, Rome, Italy; 1997.

32. Mondini L. Noorani A. Paguotta M.A. Assessing plant genetic diversity by molecular tools. Diversity. 2009; 1:19–35. http://doi.org/10.3390/d1010019

33. Kordrostami M. Rahimi M. Molecular markers in plants: concepts and applications Czech J. Genet. Plant Breed. 2002; 38(1):29–40.

34. IBPGR. Descriptors for *Xanthosoma*. International Board for Plant Genetic Resources, Rome, Italy; 1989.

35. Wild SA. Voigt GK. Munsell Color Charts for Plant Tissues. Soils Department, University of Wisconsin, Munsell Book Color, 617 Little Britain Road, New York; 1977.
36. Catherbras C. Traore C. Malapa R. Risterucci A. Chair H. characterization of microsatellites in *Xanthosoma sagittifolium* (Araceae) and cross-amplification in related species. Appl Plant Sci. 2014; 2(6); 2(6):129-134 http://doi.org/10.3732/apps.1400027
37. Currie-Fraser E. Shah P. True S. Data analysis using GeneMapper® v4.0. Comparing the Newest Generation of GeneMapper Software to Legacy Genescan® and Genotyper® Software. J Biomolec Techni. 2010. PMCID:PMC2918126
38. IBM SPSS Statistics for Windows, Version 23.0, Armonk, IBM Corp., New York; 2015.
39. SAS: Statistical Analysis Software. Version 9.3. SAS Institute, Inc., USA; 2011.
40. Kimura M. Crow J. The number of alleles that can be maintained in a finite population. Genetics. 1964; 49:725–738. PMCID: PMC1210609
41. Lewontin RC. Testing the theory of natural selection. Nature. 1972; 236: 181–182. http://doi.org/10.1038/236181a0
42. Yeh FC. Yang R. Boyle T. Popgene version 1.32: Microsoft Windows-based freeware for population genetic analysis. University of Alberta, Edmonton; 1999.
43. Peakall R. Smouse PE. GenAlEx 6.503: Microsoft Window-based freeware for population genetic analysis. Mol Ecol Notes. 2006; 6:288–295.
44. Minitab Statistical Software, Version 17, Minitab Inc., USA; 2013.
45. Felsenstein J. PHYLIP (Phylogeny Inference Package) version 3.6, distributed by the author, Department of Genome Sciences, University of Washington, USA; 2005.
46. Nei M. Tajima F. Yateno Y. Accuracy of estimated phylogenetic trees from molecular data. II. Gene frequency data. J Mol Evol. 1983; 19:150–173. http://doi.org/10.1007/BF02300753
47. Daniel D. Christian S. Microsatellite analyser (MSA): A platform independent analysis tool for large microsatellite data sets. Mol Ecol. 2003; 1:167-169. http://doi.org/10.1046/j.1471-8286.2003.00351.x
48. Opoku-Agyeman MO. Benneti-Lartey SO. Markwei C. Agro-morphological and sensory characterization of cocoyam (*X. sagittifolium* (L.) (Schott) germplasm in Ghana. Ghana J Agric Sci. 2004; 37(1):23–31. http://doi.org/10.4314/gjas.v37i1.2076
49. Villavicencio MLH. Ignacio RMA. Villancio VT. Garcia JNM. Morphological characterization and genetic diversity assessment of Gabing San Fernando (*Xanthosoma sagittifolium* L. Schott Milet) in the Philippines. Philip J Crop Sci. 2016; ISSN, 0117-463X.
50. Doungous O. Kalendar R. Adiobo A. Schulman AH. Retrotransposon molecular markers resolve cocoyam (*X. sagittifolium*) and taro (*Colocasia esculenta*) by type and variety. Euphytica. 2015; 206(2):541–554. http://doi.org/10.1007/s10681-015-1537-6
51. Geraci A. Raimondo FA Troia A. Genetic diversity and local population structure in *Ambrosina bassii* (Araceae, Ambrosineae), a Mediterranean relict species. Biochem Syst Ecol. 2009; 37:737–746. http://doi.org/10.1016/j.bse.2009.12.002
52. Acosta-Mercado D. Bird-Pico FJ. Kolterman DA. Genetic variability of *Anthurium crenatum* (L.) Kunth (Araceae) in Puerto Rico. Caribbean J Sci. 2002; 38:118–124.
53. Hedrick PW. A Standard genetic differentiation measure. Evolution. 2005; 59 (8):1633–1638. http://doi.org/10.1111/j.0014-3820.2005.tb01814.x

Supporting information

S1 Table. Passport data.

S2 Table. Selected morphological descriptors.

S3 Table. Primers sequences.

Map representing the collection Zones

Click here to access/download;Figure;Fig.1.tif

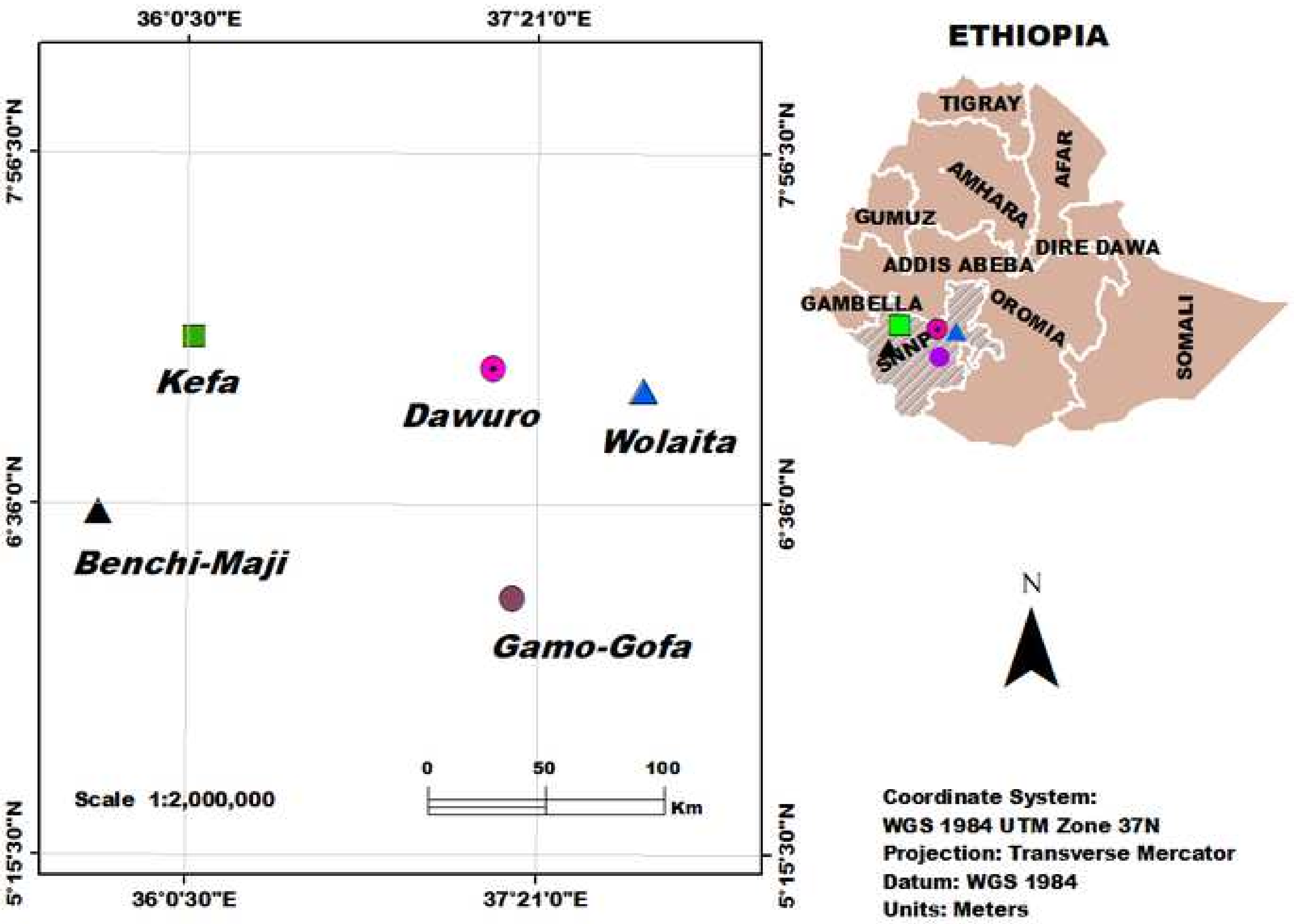

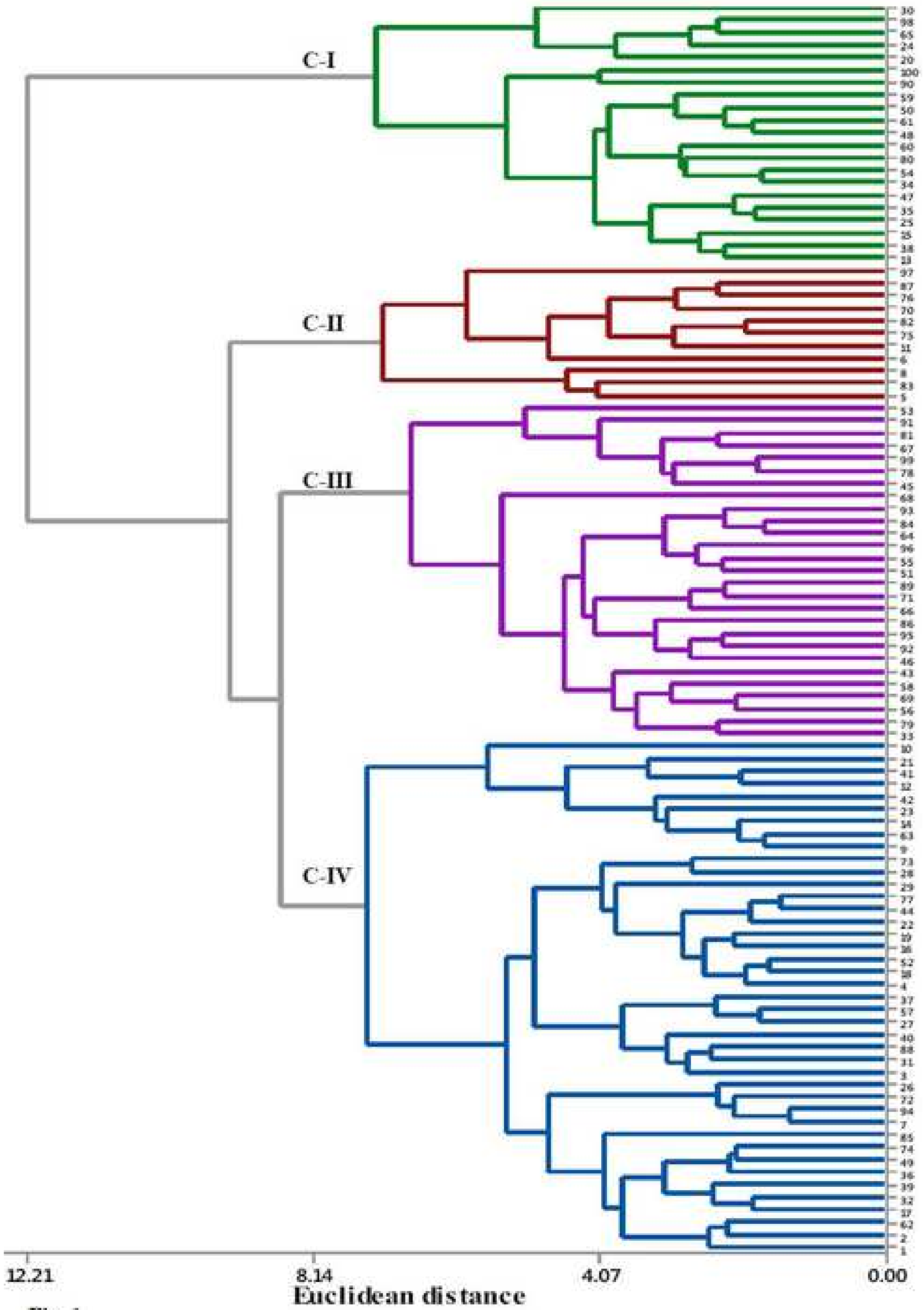

Fig. 1

Neighbour Joinings tree

Click here to access/download;Figure;Fig.3.tif

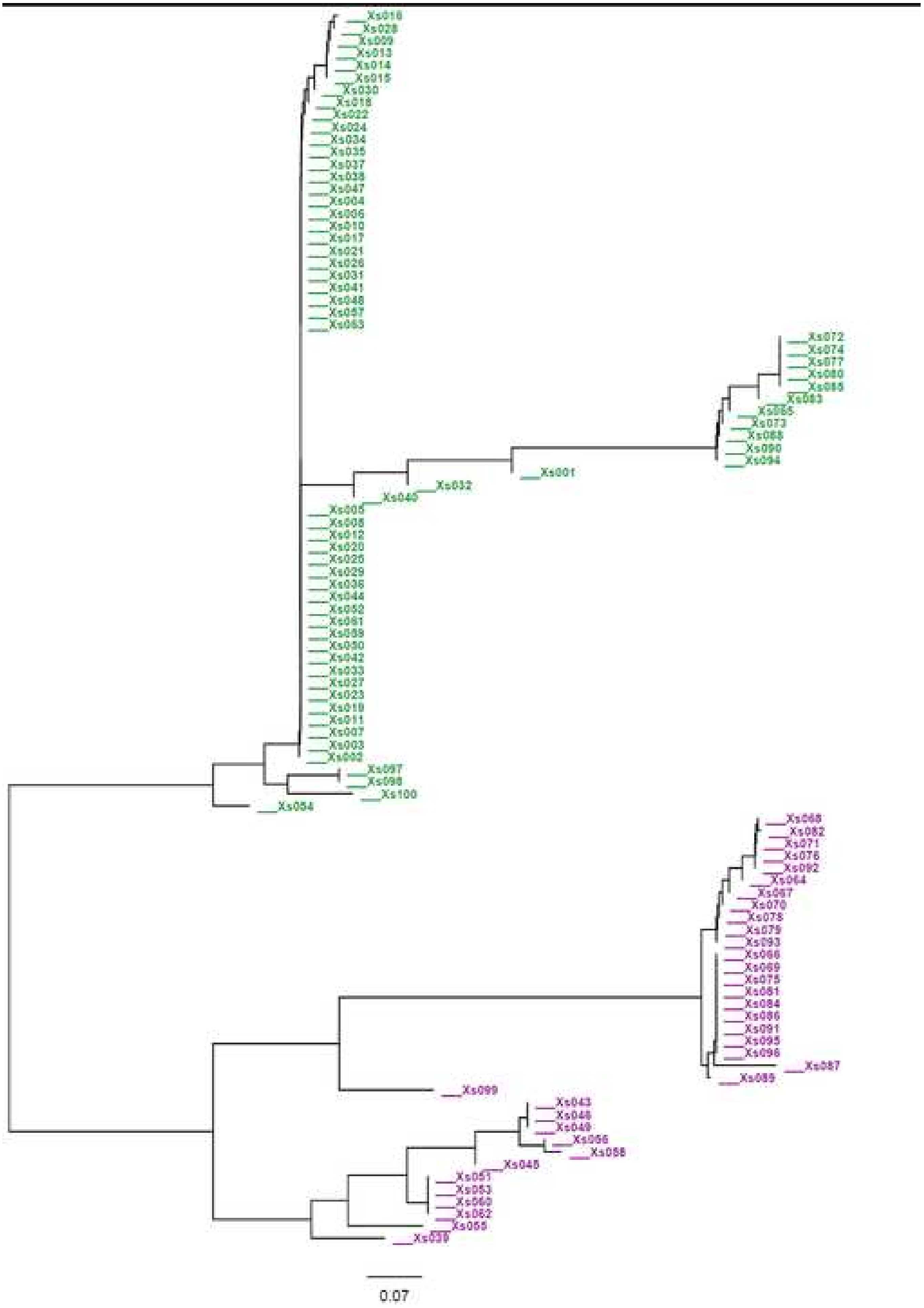

Passport data

Click here to access/download
Supporting Information
S1.docx

Selected morphological descriptors

Click here to access/download
Supporting Information
S2.docx

Primers sequences

Click here to access/download
Supporting Information
S3.docx

Genetic diversity of Ethiopian cocoyam (*Xanthosoma sagittifolium* (L.) Schott) accessions as revealed by morphological traits and SSR markers

Eyasu Wada[1]*, Tileye Feyissa[2], Kassahun Tesfaye[2], Zemede Asfaw[3] and Daniel Potter[4]

[1]Department of Biology, College of Natural Sciences, Wolaita Sodo University, Ethiopia

[2]Department of MCMB, College of Natural Sciences, Addis Ababa University, Ethiopia

[3]Department of Plant Biology and Biodiversity Management, College of Natural Sciences, Addis Ababa University, Ethiopia

[4]Department of Plant Sciences, College of Agricultural and Environmental Sciences, University of California-Davis, United States of America

*Corresponding author's email: eyasuwada@gmail.com (EW)

Abstract

Cocoyam (*Xanthosoma sagittifolium* (L.) Schott) originated and first domesticated in tropical America. It is exotic to Ethiopia, but widely cultivated for its edible cormels and leaves. There is a dearth of information on the genetic diversity of Ethiopian cocoyam. In order to evaluate and select cocoyam germplsam for breeding and conservation, genetic diversity of 100 Ethiopian cocoyam accessions (65 green- and 35 purple- cocoyam) were analyzed using 29 morphological traits (16 qualitative and 13 quantitative) and 12 SSR loci. Two classes of qualitative traits were observed. ANOVA revealed significant variation in 11 (84.6%) of the 13 studied quantitative traits. The SSR marker analysis showed high genetic diversity. A total of 36 alleles were detected with range 2 to 5 alleles (an average of 3.273 alleles per locus). The average observed heterozygosity (Ho) and expected heterozygosity (He) values across populations were, 0. 503 and 0.443, respectively. The analysis of molecular variance showed that the variation among population explained 14%, while the variation from among individuals within populations and within individuals explained 18% and 68% of the total variation, respectively. Cluster analysis grouped the accessions irrespective of the collection sites. Dendrogram based on Nei's standard genetic distance grouped the green cocoyam accessions together while the purple cocoyam accessions occupied separate position within the dendrogram. Significant variation in quantitative traits and high level of genetic diversity revealed by SSR markers can fairly indicate that genetically diverse cocoyam accessions probably with multiple lineage might have been introduced multiple times, through multiple routes and probably by multiple agents. High genetic diversity should be considered as a good opportunity for breeding and conservation of cocoyam accessions. Further taxonomic and phylogenetic study is recommended to clarify the taxonomic group of Ethioan cocoyam accesions and to tress their evolutionary relationship with *Xanthosoma* species elsewhere.

1. Introduction

Cocoyam (*Xanthosoma sagittifolium* (L.) (Schott) is an herbaceous, monocotyledons crop in the Araceae family. Cocoyam originated and first domesticated in tropical America, where it was cultivated from very ancient times [1]. It is one of the oldest cultivated aroid in the world [2]. Nowadays, it is cultivated in many tropical countries for its edible cormels and leaves [3].

There is much confusion, discrepancies and uncertainties regarding the taxonomy of *Xanthosoma* at the species level [4]. Many names are associated with the cultivated species of the genus *Xanthosoma* [5-6] but the name *Xanthosoma sagittifolium (*L.) (Schott) has usually been given to all cultivated species of the genus [6] and several common names are also used to refer the cultivated species [5, 7-10]. In the course of this study, cocoyam is used to refere to *X. sagittifolium* , which is accepted species name of cultivated species of the genus *Xanthosoma* in the Araceae family [11] and in The Plant List [12].

Cocoyam was introduced into Eastern Africa through Western Africa [13]. In Ethiopia, cocoyam is largely unknown or considered synonymously with other related aroid, *Colocasia esculenta* (L.) Schott), which is locally known as taro or "*Godere*", [14]. However, cocoyam can be distinguished morphologically from taro by the place where the petiole meets the leaf. In cocoyam, the petiole attachment is at the margin of the leaf between the lobes. In taro, the petiole attachment is peltate or around the middle of the leaf [5].

Cocoyam flowers rarely. When occurs, the flowers are monoecious within a compound inflorescence. The female flowers are at the base of the spadix, and the male flowers are above. Sterile flowers are located between the pistillated and staminated flowers. The inflorescence is protogynous. The stigma is normally receptive two to four days before pollen shed [15]. This makes the classical breeding difficult. Cocoyam rarely set sexual seeds and hence it is mainly propagated vegetatively from corm sets, headsets or cormels [16] but

treatments were successfully applied to induce a flowering with artificial pollination techniques. Hand pollination and production of seed and seedlings has become possible at experimental settings [17-18].

Cocoyam grows in a variety of substrates and habitats in full sun or deeply shaded areas under the canopy of natural forests. It tolerates drier, but not waterlogged soils [7]. It is a lowland plant but grows in highlands [15]. It is a perennial plant but most often cultivated as an annual crop for food value. Harvest occurs during the dry season, 9-12 months after plantation [9]. At the end of the growing season, leaves die and shoots wither completely [16, 19].

Many developing countries in the tropics depend on aroids in the tribes *Colocasieae* and *Caladieae*, i.e., taro (*C. esculenta*) and cocoyam (*X. sagittifolium*) as sources of carbohydrate [20]. Cocoyam has overtaken taro as the main edible aroid in many tropical areas [3]. It is an important crop in many parts of the tropical world, mainly for smallholder farmers, in playing a major role in the lives of many as a food security crop. Both its leaves and its starch-rich cormels are eaten after cooking [2-3].

In Ethiopia, cocoyam has spread widely and become an important part of the agriculture and food systems of the local people. There is considerable amount of cocoyam gene pool in farmers' fields [21-28] but did not receive thoughtful research effort as it is a neglected crop by research and development community. The germplasms in the farmers' field are the major hope for use, conservation and improvement of the crop. Thus, collecting accessions and assessing genetic diversity are very important to identify germplasms for efficient and effective usage, conservation and improvement.

Morphological traits are conventional tools to analyze the genetic diversity by measuring the variation among accessions [29]. It is an essential step for effective utilization of germplasms because it offers a useful approach for assessing the extent of diversity [30]. The

genetic information provided by morphological traits is, however, often limited and expression of quantitative traits is subjected to strong environmental influence [31-32]. Assessing genetic diversity of plant species by using molecular markers has several advantages over morphological traits based characterization [33] although they cannot completely replace it. Complementary to morphological traits based characterization, the use of molecular markers has been importantly increasing [31]. This study employed genetic characterization using morphological descriptors and SSR molecular markers to assess the genetic diversity of Ethiopian cocoyam (*Xanthosoma sagittifolium* (L.) Schott) accessions to enhance effective usage, conservation and improvement of cocoyam.

2. Materials and methods

2.1 Plant materials

Similar sized cormel germplasms of 100 cocoyam accessions were sampled from 5 zones (Bench-Maji, Kefa, Dawuro, Wolaita and Gamo-Gofa) (Fig. 1). Twelve to twenty-eight accessions were sampled from each zone (S1Table). The individual accessions were collected from at least 5 km apart unless they were clearly distinguished by leaf and petiole color difference (65 green-cocoyam and 35 purple-cocoyam). The accessions that were collected from the same zone were considered as one population for the genetic diversity studies in assumption that cormel germplasms were more likely shared within zones than among zones. The same language is spoken within the zones and there are frequent local markets within zones, where local people buy and sell commodities. The accessions are kept as living material at the Areka Agricultural Research Center.

Fig. 1 Map representing the collection zones of the cocoyam accessions. Twelve to twenty-eight accessions were sampled from each Zone (S1 Table). The individual accessions were collected from at least 5 km apart unless they were clearly distinguished by leaf and petiole color difference.

2.2 Experimental design and crop management

The cormel germplasms (100) were planted in a common research garden at Areka Agricultural Research Center, 303 km southwest of Addis Ababa, Ethiopia in 10*10 simple lattice square design in well-drained, loose soil on flat ground in 2016 cropping season. Five cormels of an accession were planted in a single row plot of 3 m, spaced 0.6 m between plants and 0.75 m between rows. Weeding was conducted as required to keep plots weed free. Similar sized cormels were harvested at the end of the 12th month after the date plantation and the experiment was repeated in 2017 cropping season using the same experimental design and taking similar crop management practices.

2.3 Morphological traits and data collection

Formatted: Font: 16 pt

Twenty-nine morphological traits (S2 Table) were selected from International Board for Plant Genetic Resources Descriptors for *Xanthosoma* [34]. The qualitative data were scored for qualitative traits such as plant growth habit, petiole attachment, petiole color (upper 2/3rd), petiole color (lower 1/3rd), color of the edge of petiole, lamina orientation, leaf margin color, leaf shape, color of upper leaf surface, color of lower leaf surface, position of cormel apex, shape of cormels, color of cormel apex and fresh cormel color at the end of the 7th month after the date of plantation, from the middle individual plant. The Munsell Plant Tissue Color Chart [35] was used to discriminate colors. The quantitative data were recorded from the middle three plants of each plot, leaving the two plants that grown as boarder plants. The aboveground quantitative traits such as overall plant height (cm), petiole length (cm), petiole sheath length (cm), circumference of the pseudo-stem (cm), lamina length (cm) and lamina width (cm) were measured at the end of the 7th month after the date of plantation. The underground quantitative traits such as the number of cormels/plant, cormel length (cm), cormel diameter (cm), cormel fresh weight/plant (kg), corm length (cm), corm diameter (cm)

and corm fresh weight/plant (kg) were recorded at the end of 11th months after date of plantation.

2.4 DNA extraction

Formatted: Font: 16 pt

Young leaf samples were collected by using silica gel (Bio lab). Total genomic DNA was extracted using DNeasy plant min kit (QIAGEN) according to the manufacture's instruction. The DNA was checked on a 2% agarose gel. The quantity and quality of DNA were assessed by using Nanodrop-2000.

2.5 SSR primers and PCR amplification

Formatted: Font: 16 pt

Seventeen microsatellite primer pairs identified by Cathebras et al. [36] for *X. sagittifolium* were tested with 5 randomly selected samples. Twelve SSR primer pairs (S3 Table), which gave an intense band on an agarose gel, were selected for subsequent analysis. The forward primer of each of the 12 pairs was labeled either with 6-FAM (6-Carboxyfluorescein) or Hexachloro-fluorescein (HEX). The markers were paired into 6 PCR sets, each set containing 6-FAM labeled primer pair and HEX labeled primer pair. These labeled primers were used to test 15 samples in a pre-analysis.

PCR was performed in a total volume of 20 µL containing 2 µL of 10x PCR buffer (15 mM $MgCl_2$ included), 0.4 µL dNTP mix (10 mM each), 0.5 µL (16 pmol/µL) of each of the forward and reverse SSR primers, 2 µL genomic DNA (20 ng/µL), 0.2 µL Taq polymerase and 14.4 µL ddH_2O. Amplification was performed in ABI 2720 Thermocyler. After initial denaturation at 95 °C for 2 min, 30 cycles denaturation at 94 °C for 30 s, annealing at 52 °C for 45 s, extension at 72 °C for 1 min followed with a final extension of 72 °C for 10 min were used. After verification of PCR amplification through 1% agarose gel electrophoresis, capillary electrophoresis was run by mixing the aliquots of PCR product (1 µLµl/well) with formamide (12 µlµL/well) and GeneScan™ 400HD ROX™ Size Standard (0.4 µLµl/well) in a 96 plate. The contents were denatured using a Boekel dry bath, which was heated to 95 °C

for 5 min. Then, the DNA was quenched at -20 °C for 5 min and electrophoresed on an ABI 3130XL Genetic Analyzer (Applied Biosytems). The number and sizes of electrophoresed DNA fragments were identified using GeneMapper version 4.0 [37].

2.6 Data analysis

Formatted: Font: 16 pt

Frequency distribution of qualitative traits and basic statistical parameters based on quantitative morphological traits were calculated using SPSS version 23 [38]. Analysis of variance (ANOVA) was computed by PROC GLM procedure of SAS 9.3 [39] because the relative efficiency of the lattice design compared with CRBD was low (less than 105%). The number of alleles (Na), the number of effective allele (Ne) [40], Shanon information index (I) [41], observed (Ho) and expected (He) heterozygosity within individual populations, were generated using the program POPGENE version 1.32 [42]. Analysis of molecular variance (AMOVA) was performed to evaluate the partitioning of molecular variation within and among populations using GenAlEx version 6.503 [43].

Clustering, which is an important clue for visual perception was constructed morphological as well as SSR marker data. The quantitative morphological traits were subjected to a cluster analysis using Minitab17.1 [44], by employing average linkage clustering strategy of the observation. Variables were standardized to a common scale by subtracting the means and dividing by the standard deviation. The number of clusters was determined by following the steps recommended by Minitab 17.1 [44]. The dendrogram showing the Euclidean distance between clusters was constructed by plotting the results of cluster analysis using the same program. The genetic clustering based on SSR data was assessed by performing using neighbor joining algorithm implemented in the computer program PHYLIP version 3.6 [45] using Nei's genetic distance [46] based on the frequencies generated by using MSA version 4.05 [47].

3.Results

3.1 Qualitative traits

Formatted: Font: 16 pt

Of 16 qualitative traits, 9 discriminated the cocoyam accessions into two groups, where 35 purple cocoyam accesions were grouped together and 65 green cocoyam accessions were occupied another group.Two classes of petiole color (upper $2/3^{rd}$), petiole color (lower $1/3^{rd}$), color of edge of petiole sheath, lamina orientation, color of upper leaf surface, color of lower leaf surface, color of veins on lower leaf surface, position of color apex and shape of cormels existed in the accessions. Qualitative traits such as plant growth habit, petiole attachment, leaf shape, leaf margin color, color of veins on upper leaf surface, color of cormel apex and fresh cormel color did not discriminate the accessions included in this study (Table 1).

Table 1. Frequency distribution of 16 qualitative traits of cocoyam accessions.

Formatted Table

No	Plant character	Character state	%*
1	Plant growth habit	Acaulescent	100
2	Petiole attachment	Non-peltate	100
3	Petiole color (upper $2/3^{rd}$)	Green	65
		Purple	35
4	Petiole color (lower $1/3^{rd}$)	Green streaked with purple	65
		Purple	35
5	Color of edge of petiole sheath	The same as the rest of petiole and sheath	35
		Purple	65
6	Lamina orientation	One plane - apex down (Droopy)	35
		3-dimentional (cup-shaped)	65
7	Leaf shape	Sagitate basal lobes	100
8	Leaf margin color	Purple edge	100
9	Color of upper leaf surface	Medium green	65
		Dark green	35
10	Color of lower leaf surface	Light green	65
		Purplish green	35
11	Color of veins on upper leaf surface	Lighter green than lamina	100
12	Color of veins on lower leaf surface	Same as color as lamina	65
		Purple	35
13	Position of color apex	Underground	65
		Above ground and under ground	35
14	Shape of cormels	Globose	65
		Ovate	35
15	Color of cormel apex	Red	100
16	Internal color of cormels	White streaked with purple	100

*100% indicates the character common to both green leaf/petiole-cocoyam and purple leaf petiole-cocoyam accesions, 65% character specific to green- cocoyam accessions and 35% character specific to purple- accessions.

3.2 Descriptive statistical parameters and variance

Formatted: Font: 16 pt

Descriptive statistics showed that the mean values above ground quantitative traits such of plant height, petiole length (cm) petiole sheath length (cm), lamina length (cm) lamina width (cm) and circumference of pseudo-stem (cm) were 71.49, 55.22, 31.24, 37.77, 22.02 and 28.83, respectively. The mean values of underground quantitative traits such as the number of cormels/plant, cormel length (cm), cormel diameter (cm), cormel fresh weight/plant (kg), corm length (cm), corm diameter (cm) and corm fresh weight/plant (kg), respectively, were 10.05, 9.05, 3.26, 1.28, 11.94, 5.70 and 1.18. Maximum SD (5.63) corresponded to plant height and minimum SD (0.31) corresponded to cormel fresh weight/plant (kg). The coefficient of variation (CV %) varied from 7.65% for lamina width to 29.10% for the corm fresh weight/plant. Analysis of variance (ANOVA) revealed significant variation in 11(84.6%) of the 13 studied quantitative traits (Table 2).

Table 2. Basic statistics and summary of mean squares of quantitative traits

Quantitative traits	Mean	SD	Minimum	Maximum	CV%	Mean squares		
						Rep (df =1)	Accessions (df =99)	Error (df =81)
PH	71.49	5.63	57.58	84.83	7.87	100.30	82.46*	54.46
PL	55.22	4.82	42.75	67.83	8.72	294.64	49.57*	31.66
PSL	31.24	3.46	25.50	43.92	11.08	333.62	28.16	21.48
LL	37.77	3.20	29.69	45.25	8.48	20.09	16.73***	8.10
LW	22.02	1.68	17.28	25.47	7.65	0.74	4.96***	2.41
CPS	28.83	2.78	21.42	34.42	9.66	128.27	14.02***	5.72
CN	10.05	1.81	5.92	15.33	17.98	5.01	7.40**	3.91
CL	9.05	1.09	6.58	11.83	12.00	0.02	2.17	2.30
CD	3.26	0.46	2.25	4.21	13.97	1.12	0.29**	0.17
CFW	1.28	0.31	0.68	2.42	23.79	1.71	0.23**	0.13
CrL	11.94	2.11	3.49	16.92	17.71	102.84	7.65**	4.38
CrD	5.70	0.77	3.49	7.73	13.52	2.95	1.06***	0.45
CrFW	1.18	0.34	0.53	2.30	29.10	1.98	0.24***	0.12

PH- plant height (cm), PL-petiole length (cm), PSL-petiole heath length (cm), LL-lamina length (cm), LW-lamina width (cm), CPS-circumference of pseudo-stem (cm), CN- cormel Number per plant, CL-cormel length (cm), Cormel Diameter (cm), CFW-cormel fresh weight per plant (kg), CrL-corm length (cm), CrD-corm diameter (cm), CrFW-corm fresh weight/plant(kg), df - degree of freedom, *, ** and *** significant at p= 0.05, 0.01 and 0.001, respectively.

Table 2. Basic statistics of 13 quantitative traits of cocoyam.

Quantitative traits	Mean	SD	Minimum	Maximum	CV%
Plant height (cm)	71.49	5.63	57.58	84.83	7.87
Petiole length (cm)	55.22	4.82	42.75	67.83	8.72
Petiole sheath length (cm)	31.24	3.46	25.50	43.92	11.08
Lamina length (cm)	37.77	3.20	29.69	45.25	8.48
Lamina width (cm)	22.02	1.68	17.28	25.47	7.65
Circumference of pseudo-stem (cm)	28.83	2.78	21.42	34.42	9.66
Number of cormels/plant	10.05	1.81	5.92	15.33	17.98
Cormel length (cm)	9.05	1.09	6.58	11.83	12.00
Cormel diameter (cm)	3.26	0.46	2.25	4.21	13.97
Cormel fresh weight/plant (kg)	1.28	0.31	0.68	2.42	23.79
Corm length (cm)	11.94	2.11	3.49	16.92	17.71
Corm diameter (cm)	5.70	0.77	3.49	7.73	13.52
Corm fresh weight/plant(kg)	1.18	0.34	0.53	2.30	29.10

Formatted Table

Table 3. Summary of mean squares of 13 quantitative traits of cocoyam.

Quantitative traits	Mean squares		
	Rep (df =1)	Accessions (df =99)	Error (df =81)
Plant height (cm)	100.30	82.46*	54.46
Petiole length (cm)	294.64	49.57*	31.66
Petiole sheath length (cm)	333.62	28.16	21.48
Lamina length (cm)	20.09	16.73***	8.10
Lamina width (cm)	0.74	4.96***	2.41
Circumference of pseudo-stem (cm)	128.27	14.02***	5.72
Number of cormels/plant	5.01	7.40**	3.91
Cormel length (cm)	0.02	2.17	2.30
Cormel diameter (cm)	1.12	0.29**	0.17
Cormel fresh weight/plant (kg)	1.71	0.23**	0.13
Corm length (cm)	102.84	7.65**	4.38
Corm diameter (cm)	2.95	1.06***	0.45
Corm fresh weight/plant (kg)	1.98	0.24***	0.12

df - degree of freedom, *, ** and *** significant at p= 0.05, 0.01 and 0.001, respectively.

3.3 Genetic diversity of cocoyam as revealed by SSR markers

Formatted: Font: 16 pt

Among a total of 12 SSR loci, 11 (91.7%) showed polymorphism. A total of 36 alleles, ranged from 2 to 5 per locus with an average of 3.273, were detected (Table 3). The Ne per locus ranged from 1.412 (locus mXsCIR07) to 3.759 (locus mXsCIR22) with mean Ne of 2.516. The observed heterozygosity (Ho) ranged from 0.000 (loci mXsCIR19, mXsCIR16 and mXsCIR24) to 1.000 (loci mXsCIR22 and mXsCIR27) with mean Ho of 0.508. The mean expected heterozygosity (He) was 0.566.

Table 3. Genetic diversity parameters in 100 cocoyam accessions

Locus name	Na	Ne	I	Ho	He
mXsCIR05	4	3.499	1.304	0.800	0.720
mXsCIR07	2	1.412	0.468	0.355	0.294
mXsCIR19	2	1.851	0.652	0.000	0.462
mXsCIR10	4	2.810	1.087	0.905	0.648
mXsCIR21	4	2.498	1.098	0.372	0.603
mXsCIR22	4	3.759	1.354	1.000	0.738
mXsCIR11	4	2.158	0.969	0.650	0.539
mXsCIR24	2	1.821	0.643	0.000	0.453
mXsCIR12	3	2.368	0.972	0.505	0.581
mXsCIR27	5	3.689	1.364	1.000	0.733
mXsCIR16	2	1.807	0.639	0.000	0.449
Mean	3.273	2.516	0.959	0.508	0.566
SD	1.103	0.823	0.318	0.395	0.142

Na - number of different alleles, Ne - effective number of alleles, I - Shannon information index, Ho- observed heterozygosity and He - expected heterozygosity.

Formatted Table

3.4 Genetic diversity within populations

Formatted: Font: 16 pt

The observed number of alleles (Na), effective number of alleles (Ne), observed heterozygosity (Ho) and expected heterozygosity (He) and Shannon information index (I) across populations are presented in Table 5.The The observed number of alleles (Na) varied from 1.727 (Bench-Maji, Kefa) to 3.091 (Gamo-Gofa) and the effective number of alleles (Ne) varied from 1.560 (Kefa) to 2.660 (Gamo-Gofa), with mean Na and Ne across populations were 2.455 and 2.091, respectively. The expected heterozygosity (He) values ranged from 0.267 (Bench-Maji) to 0.608 (Gamo-Gofa), mean He was 0.443. Shannon diversity index reflected similar trend of He (Table 4). The partitioning of molecular variance (AMOVA) showed that the highest genetic variability (68%) existed within individuals and the lowest variability (14%) existed among populations (Table 65).

Table 4. Genetic diversity within populations.

Population	N	Na	Ne	Ho	He	I
Bench-Maji	14	1.727	1.631	0.446	0.267	0.391
Kefa	12	1.727	1.560	0.515	0.289	0.399
Dawuro	28	2.818	2.093	0.492	0.476	0.787
Wolaita	28	2.909	2.513	0.520	0.574	0.935
Gamo-Gofa	18	3.091	2.660	0.540	0.608	0.990
Average across populations	20	2.455	2.091	0.503	0.443	0.700
At species level	100	3.273	2.516	0.508	0.566	0.959

N - number of accessions; Na - number of different alleles; Ne - effective number of alleles; Ho- observed heterozygosity; He - expected heterozygosity; I - Shannon information index.

Table ~~6~~5. Summary of AMOVA for five cocoyam populations based on SSR markers

Source of variation	df	Sum of squares	Estimated variance	% of variation	Fst	P value
Among Pops	4	94.046	0.510	14	0.143	0.001
Among Indivs	95	349.994	0.637	18		
Within Indivs	100	241.000	2.410	68		
Total	199	685.040	3.558			

3.5 Clustering of accessions

The cluster analysis based on the mean values of 13 quantitative morphological traits grouped the 100 cocoyam accessions into 4 clusters (Fig. ~~1~~2). The clustering means of accessions based on 13 quantitative traits is presented in Table ~~7~~6. Cluster four (C-IV) was the largest cluster composed of 41 (41%) of accessions and the least compacted cluster than all other clusters, having the largest within cluster sum of squares. Accessions in this cluster were characterized by the highest mean value for number of cormels/plant. Cluster three (C-III) was the second larger cluster contained 27 (27%) of the total accessions. Of the 27 accessions grouped in this cluster, 25 (92.3%) were purple-cocoyam accessions. Cluster two (C-II) included the smallest number of accessions (11%) than all other clusters and it is a more compact cluster, which has the smallest within cluster sum of squares. Accessions in this cluster were characterized by the highest mean values for plant height, petiole length, lamina length and width, circumference of pseudo-stem and corm length. Cluster one (C-I) contained 21 (21%) accessions, which were characterized by the lowest mean values for most of the

Formatted: Font: 16 pt

Formatted: Line spacing: Double

traits studied. The cluster analysis showed that most of the accessions collected from different zones (populations) were clustered together. Individual accessions clustering based on SSR data is presented in the neighbor joining (NJ) tree based on Nei's genetic distance. The clustering fully separated green- cocoyam accessions from the purple- cocoyam accessions (Fig. 32).

Table 76. Cluster means of 13 quantitative traits of cocoyam.

Quantitative traits	Means values of cluster			
	**C-I**	**C-II**	**C-III**	**C-IV**
PH	67.26	80.00	67.60	73.93
PL	50.16	63.16	55.15	55.51
PSL	38.44	37.86	30.80	31.21
LL	34.50	41.13	39.71	37.28
LW	20.33	23.89	22.63	22.14
CPS	26.00	31.69	30.42	28.48
CN	9.19	10.32	9.28	10.93
CL	9.03	9.61	8.60	9.21
CD	2.91	3.59	3.68	3.08
CFW	1.02	1.43	1.31	1.35
CrL	10.27	12.30	14.08	11.28
CrD	5.00	6.19	6.31	5.55
CrFW	0.92	1.29	1.51	1.07
Within cluster sum of squares	167.63	117.39	192.94	304.46

PH- plant height (cm), PL-petiole length (cm), PSL-petiole heath length (cm), LL-lamina length (cm), LW-lamina width (cm), CPS-circumference of pseudo-stem (cm), CN- cormel Number per plant, CL-cormel length (cm), Cormel Diameter (cm), CFW-cormel fresh weight per plant (kg), CrL-corm length (cm), CrD-corm diameter (cm) and CrFW-corm fresh weight/plant(kg).

Formatted Table

Fig. 21 Cluster analysis showing the relationship among cocoyam accessions based on quantitative traits. The numbers stand for the serial numbers of the accession code. The accessions 1-14, 15-26, 27-54, 55-82 and 83-100 were from Bench-maji, Kefa, Dawuro, Wolaita and Gamo-Gofa zones, respectively.

Fig. 2 3 Neighbour Joinings tree showing the relationships among Ethiopian cocoyam accessions. The tree was generated based on Nei's genetic distance using 11 microsatellite markers. Green- and purple- represent green- and purple- cocoyam accessions. The accessions Xs001-Xs014, Xs015-Xs026, Xs027-Xs054, Xs055-Xs082 Wolaita and Xs083 -Xs100 were from Bench-maji, Kefa, Dawuro, Wolaita and Gamo-Gofa zones, respectively.

4. Discussion

This study employed the techniques of genetic characterization using morphological traits and SSR molecular markers, which provided insight into a broader context for the genetic diversity and clustering of cocoyam accessions from Ethiopia. The majority (84.6%) of the quantitative morphological traits showed significant variation among accessions. Similar to this finding other authors, who had assessed genetic diversity of cocoyam accession by using morphological traits also reported significance variation among the studied accessions [24, 48-49]. SSR markers revealed high genetic diversity across populations. Most of (91.7%) the SSR loci were polymorphic within cocoyam accessions. In related study, Doungous et al. [50] reported 88% to 97% polymorphic loci across 20 cocoyam accessions from Cameroon using retrotransposon molecular markers. The avergae He across populations (He = 0.443) was higher than that of *Ambrosina bassii* L. (Araceae, Ambrosineae) (He = 0.263) [51] and *Anthurium crenatum* (L.) Kunth (Araceae) (He = 0.167) [52], implying that our collections contained genetically diverse cocoyam accessions. The observed heterozygosity was very high (Ho = 1) for two loci (mXsCIR22 and mXsCIR27) (Table 3) and six SSR markers presented Ho values higher than He values. SSR markers identified by Cathebras et al. [36], to assess cocoyam genetic diversity, gave variable degrees of heterozygosity, observed at levels ranging from 0.000 to 0.970. This excess in heterozygotes might be due to high rates of asexual reproduction in cocoyam due to vegetative propagation. The inflorescence of cocoyam is protogynous[15].

The extent of genetic clustering is of great interest in a number of biological fields including evolution, conservation and breeding [53]. The qualititative morphological traits and SSR markers-based clustering analyses effectively separated the purple cocoyam accessions from green cocoyam accessions. Cluster analysis based on quantitative morphological data also grouped most of (92.3%) the purple cocoyam accessions in distinct

cluster (cluster III), suggesting the existence of genetic difference between green-and purple-cocoyam accessions. The color difference could be considered as important for easy identification of accessions. Most of the accessions collected from different zones (populations) were collected from different zones, were grouped regardless of the collection zones, indicating that there have been movement of germplasm among zones , possibly by farmer-to-farmer planting materials exchange. Previously, a similar study [26] showed that most of the cocoyam accessions from different districts and villages of Ethiopia were clustered together.

For the reason that the exact date of cocoyam introduction into Ethiopia and who introduced it cannot be unequivocally spelt out, high genetic diversity among cocoyam accession and a significant differentiation between the morphotypes (green- and purple cocoyam) can fairly indicate that or the mixing gentically distinic morphotypes, which may represent different species or cultivars of *Xanthosoma* .geneticaly diverse cocoyam accessions probably with multiple lineage might have been introduced into the country multiple times, through multiple routes and probably by multiple agents. High genetic diversity should be considered as a good opportunity for breeding, conservation and improvement of cocoyam. Further taxonomic and phylogenetic study is recommended to clarify the taxonomic group of cocoyam landraces of Ethiopia and to tress their evolutionary relationship with *Xanthosoma* species elsewhere.

Acknowledgement

The authors appreciate Dr. Asfaw Kifle for the assistance during the field works and during morphologicla data collection.

References

1. Giacometti DC, Leon J. Tannia, yautia (*Xanthosoma sagittifolium*). In: Hernandez-Bermejo L, editor. Neglected Crops 1492 from Different Perspectives. Food and Agricultural Organization of United the United Nations, Rome, Italy; 1994. pp. 255–258.
2. Purseglove JW. Tropical crops. Monocotyledons. Longman, London, UK; 1972.

3. Ramawat KG, Merillon JM. Bulbous Plants Biotechnology. CRC press, Taylor and Francis group, USA; 2014.
4. Saborio F. Cocoyam (*Xanthosoma sagittifolium* (L.) Schott). In: Ochatt S and Jain SM editors. Breeding of Neglected and Underutilized Crops, Spices and Herbs, pp. Science Publishers, USA; 2007. pp.172-189.
5. Mayo SJ, Bogner J, Boyce PC. The Genera of Araceae. European Union by Continental Printing, Belgium; 1997.
6. Quero-Garcia J, Ivancic Q, Lebot V. Taro and Cocoyam. In: Bradishaw JE, editor. Hand Book of Plant Breeding: Root and Tuber Crops. Springer Science and Business Media, London, UK; 2010. pp. 149–172.
7. Morton JF. Cocoyams (*Xanthosoma caracu*, *X. atrovirens* and *X. nigrum*), ancient root and leaf vegetables, gaining in economic importance. Flori Stat Hortic Socie. 1972; 85: 85–94.
8. Bown D. Aroids: Plants of the Arum Family, Second ed. Timber Press, Portland Oregon, USA; 2000.
9. Raemaekers RH. Crop Production in Tropical Africa. Ministry of Foreign Affairs, External Trade and International Co-operation, Directorate General for International Co-operation, Brussels, Belgium; 2001.
10. Lebot V. The Tropical Root and Tuber Crops: Cassava, Sweet Potato, Yams and Aroids. Crop Production Science in Horticulture Series No 17, CABI, UK; 2009.
11. Govaerts R, Frodin DG, Bogner J. World checklist and bibliography of Araceae (and Aroraceae). Royal Botanic Gardens, UK; 2002.
12. The Plant List (http://www.theplantlist.org).
13. Maundu P, Achigan-Dako E, Morimoto Y. Biodiversity of African Vegetables. In: Shackleton CM, Pasquini M, Drescher AW, editors). African Indigenous Vegetables in Urban Agriculture, Earthscan, UK; 2009. pp. 65 –105.
14. Simone A. Taro roots in North Omo. FPR Technical Pamphlet No. 2, Addis Ababa, Ethiopia; 1992.
15. Manner HI. Farm and forestry production and marketing profile for Tannia (*Xanthosoma* spp.). In: Elevitch CR, editor. Specialty Crops for Pacific Island Agroforestry. Permanent Agriculture Resources, Hawaii, USA; 2011. pp. 1-16.
16. Jackson GV. Major aroids. In: Dulloo ME. Thormann I. Jorge MA. Hanson J, editors. Crop Specific Regeneration Guidelines. CGIAR System-Wide Genetic Resource Programme, Rome, Italy; 2008. pp. 1-16.
17. Wilson JE. Promotion of flowering and production of seed in cocoyam (*Xanthosoma* and *Colocasia*). International Symposium on Tropical Root and Tuber Crops, IITA, Ibadan, Nigeria; 1979.
18. Goenaga R. Hepperly P. Flowering induction, pollen and seed viability and artificial hybridization of taniers (*Xanthosoma* spp.). J Agric Uni Puerto Rico. 1990; 74(3): 253–260.
19. Onwueme IC. Charles WB. Tropical Root and Tuber Crops: Production, Perspectives and Future Prospects. Agricultural Organization of United the United Nations, Rome, Italy; 1994.

20. Goenaga R. Singh U. Beinroth FH. Prasad H. Substore-Aroid: A model in the making. Agrobiotechnology transfer 1991. 14: 1-4.
21. Asfaw Z. Origin and evolution of rural home-garden in Ethiopia. In: Friis I, editor, Biodiversity Research in the Horn of Africa Region. University of Copenhagen, Denmark; 2001. pp. 273–286.
22. Asfaw Z. Home gardens in Ethiopia: Some observations and generalizations In: Watson JW. Eyzaguirre PB, editors. Home Gardens and *in situ* Conservation of Plant Genetic Resources in Farming Systems. Proceedings of the Second International Home-Gardens Workshop, Witzenhausen, Germany; 2001. pp.125–139.
23. Nebiyu A. Awas T. Exploration and collection of root and tuber crops in Southwestern Ethiopia: its implication for conservation and research, in: Proceeding of the 11th Conference of the Crop Science Society of Ethiopia, 26-28 April, 2004, Addis Ababa, Ethiopia; 2006.
24. Nebiyu A. Gardew W. Tofu A. Abebe W. Kifle A. Etissa E. Variety development for other root and tuber crops (taro, cassava and yam). In: Woldegiorgis G. Gebre E. Lemaga B, editors. Root and Tuber Crops: The Untapped Resources. EIAR, Addis Ababa, Ethiopia; 2008. pp. 303–316.
25. Fujimoto T. Taro (*Colocasia esculenta* (L.) Schott) cultivation in vertical wet-dry environments: Farmers' techniques and cultivar diversity in southwestern Ethiopia. Econ Bot. 2009; 63(2):152–166. http://doi.org/10.1007/s12231-009-9074-7
26. Fantaw F. Nebiyu A. Mulualem T. Genetic diversity of Tannia (*X. sagittifolium* (L.) Schott) genotypes using multivariate analysis at Jimma, South west Ethiopia. Int J Plant Breed Genet. 2014; 8(4):194–204. http://doi.org/10.3923/ijpbg.2014.194.204
27. Fantaw S. Nebiyu A. and Mulualem T. Estimate of genetic components of yield and yield related traits of tannia (*Xanthosoma sagittifolium* (L.) Schott) genotypes at Jimma, Southwest Ethiopia. Afr J Agric Res. 2014; 10(1):23–30. http://doi.org/10.5897/AJAR2014.8997
28. Woldeyes F. Asfaw Z. Demisew S. Bernard R. Home-gardens of the Basketo People of southwestern Ethiopia: Sustainable agro-ecosystems characterizing a traditional landscape. Ethnobot Res Appl. 2016; 14:549–563. http://doi.org/10.17348/era.14.0.549-563
29. Sinha S. Kumaravadivel N. Understanding genetic diversity of sorghum using quantitative traits. Scientifica. 2016. http://doi.org/10.1155/2016/3075023
30. Koorneef M. Stam P. Changing paradigms in plant breeding. Plant Physiol. 2001; 125:156–159. http://doi.org/10.1104/pp.125.1.156
31. Karp A. Kresovich S. Bhat K.V. Ayad W.G. Hodgkin T. Molecular tools in plant genetic resources conservation: a guide to the technologies. International Plant Genetic Resources Institute, Rome, Italy; 1997.
32. Mondini L. Noorani A. Paguotta M.A. Assessing plant genetic diversity by molecular tools. Diversity. 2009; 1:19–35. http://doi.org/10.3390/d1010019
33. Kordrostami M. Rahimi M. Molecular markers in plants: concepts and applications Czech J. Genet. Plant Breed. 2002; 38(1):29–40.
34. IBPGR. Descriptors for *Xanthosoma*. International Board for Plant Genetic Resources, Rome, Italy; 1989.

35. Wild SA. Voigt GK. Munsell Color Charts for Plant Tissues. Soils Department, University of Wisconsin, Munsell Book Color, 617 Little Britain Road, New York; 1977.
36. Catherbras C. Traore C. Malapa R. Risterucci A. Chair H. characterization of microsatellites in *Xanthosoma sagittifolium* (Araceae) and cross-amplification in related species. Appl Plant Sci. 2014; 2(6); 2(6):129-134 http://doi.org/10.3732/apps.1400027
37. Currie-Fraser E. Shah P. True S. Data analysis using GeneMapper® v4.0. Comparing the Newest Generation of GeneMapper Software to Legacy Genescan® and Genotyper® Software. J Biomolec Techni. 2010. PMCID:PMC2918126
38. IBM SPSS Statistics for Windows, Version 23.0, Armonk, IBM Corp., New York; 2015.
39. SAS: Statistical Analysis Software. Version 9.3. SAS Institute, Inc., USA; 2011.
40. Kimura M. Crow J. The number of alleles that can be maintained in a finite population. Genetics. 1964; 49:725–738. PMCID: PMC1210609
41. Lewontin RC. Testing the theory of natural selection. Nature. 1972; 236: 181–182. http://doi.org/10.1038/236181a0
42. Yeh FC. Yang R. Boyle T. Popgene version 1.32: Microsoft Windows-based freeware for population genetic analysis. University of Alberta, Edmonton; 1999.
43. Peakall R. Smouse PE. GenAlEx 6.503: Microsoft Window-based freeware for population genetic analysis. Mol Ecol Notes. 2006; 6:288–295.
44. Minitab Statistical Software, Version 17, Minitab Inc., USA; 2013.
45. Felsenstein J. PHYLIP (Phylogeny Inference Package) version 3.6, distributed by the author, Department of Genome Sciences, University of Washington, USA; 2005.
46. Nei M. Tajima F. Yateno Y. Accuracy of estimated phylogenetic trees from molecular data. II. Gene frequency data. J Mol Evol. 1983; 19:150–173. http://doi.org/10.1007/BF02300753
47. Daniel D. Christian S. Microsatellite analyser (MSA): A platform independent analysis tool for large microsatellite data sets. Mol Ecol. 2003; 1:167-169. http://doi.org/10.1046/j.1471-8286.2003.00351.x
48. Opoku-Agyeman MO. Benneti-Lartey SO. Markwei C. Agro-morphological and sensory characterization of cocoyam (*X. sagittifolium* (L.) (Schott) germplasm in Ghana. Ghana J Agric Sci. 2004; 37(1):23–31. http://doi.org/10.4314/gjas.v37i1.2076
49. Villavicencio MLH. Ignacio RMA. Villancio VT. Garcia JNM. Morphological characterization and genetic diversity assessment of Gabing San Fernando (*Xanthosoma sagittifolium* L. Schott Milet) in the Philippines. Philip J Crop Sci. 2016; ISSN, 0117-463X.
50. Doungous O. Kalendar R. Adiobo A. Schulman AH. Retrotransposon molecular markers resolve cocoyam (*X. sagittifolium*) and taro (*Colocasia esculenta*) by type and variety. Euphytica. 2015; 206(2):541–554. http://doi.org/10.1007/s10681-015-1537-6
51. Geraci A. Raimondo FA Troia A. Genetic diversity and local population structure in *Ambrosina bassii* (Araceae, Ambrosineae), a Mediterranean relict species. Biochem Syst Ecol. 2009; 37:737–746. http://doi.org/10.1016/j.bse.2009.12.002
52. Acosta-Mercado D. Bird-Pico FJ. Kolterman DA. Genetic variability of *Anthurium crenatum* (L.) Kunth (Araceae) in Puerto Rico. Caribbean J Sci. 2002; 38:118–124.
53. Hedrick PW. A Standard genetic differentiation measure. Evolution. 2005; 59 (8):1633–1638. http://doi.org/10.1111/j.0014-3820.2005.tb01814.x

Supporting information

S1 Table. Passport data.

S2 Table. Selected morphological descriptors.

S3 Table. Primers sequences.

Response to Reviewers

First we would like to thank the reviewers for taking their time to give us these constructive comments. We have replied to the comments as follows:

- **Response to general comments**
  - Our manuscript have been carefully checked and revised as needed according to the PLOS ONE's style requirements.
  - The financial disclosure statement is changed and the cover letter is updated
  - Figure files are resubmitted carefully checking PLOS ONE requirements of figure files
  - ORCID included
  - Captions for supporting information files are included at the end of the manuscript and in text citations are updated

Reply to reviewer's comment

Reviewer #1

- **Clarify objective and elaborate hypothesis.**
  - Objective is clarified and hypothesis is elaborated
- **Provide passport data?**
  - Provided as supplementary information (S1 Table. Passport data)
- **Provide Map?**
  - Provided
- **Improve Quality of dendograms and figures?**
  - Improved
- **A reader may be confused about our study materials and feel that *Colocasia* might have been included because of a sentence in the introduction explaining that in Ethiopia the two species are considered synonymously.**
  - We have edited the sentence and clarified it to avoid any confusion
- **Size and diameter of corm?**
  - The size and diameter of *Xanthosoma* corm is 10 to 15 cm in diameter but here the average diameter of corm recorded from 3 replication for two years experiment were 4.29 to 7.73 cm and the overall mean was 5.70 cm. Such difference may be due to:

  1) Cocoyam is a perennial plant, but for practical purposes, the corm and cormles were intentionally measured separately at the end of 12th months after plantation (annually). This annual harvest could be the reason for the size and diameter reduction. Actually, we encountered larger sized corm at some fields where cocoyam grown as perennial crop when we were collecting germplasms, but which was not at research field (where it was cultivated annually).
  2) The soil type of the experimental site could be another reason.
- **One or several *Xanthosoma* species?**
  - In general, the division of the genus *Xanthosoma* into species has been difficult [1]. There is much confusion, discrepancies and uncertainties regarding the taxonomy of *Xanthosoma* at the species level. Various names have been used synonymously [2-3], the same plant being given more than one species name [4]. Thus, the name of *Xanthosoma sagittifolium* (L.) Schott has usually been given to the most cultivated members of this genus (Giacometti and [5-6]. In the course of this study, we refer to the accepted species status of *X. sagittifolium* in The Plant List [2] in the Araceae family [6], which does not tackle taxonomy in further detail.
  - There was no substantial evidence to say that there were different *Xanthosoma* species in Ethiopia although qualitative morphological traits and SSR markers clustered accession into two groups. We have a plan to do taxonomic study on *Xanthosoma* species in Ethiopia. Generally, the taxonomy of *Xanthosoma* needs to be reconstructed.

1. Saborio, F. (2007). Cocoyam (*Xanthosoma sagittifolium* (L.) Schott). In: *Breeding of Neglected and Underutilized Crops, Spices and Herbs, pp.* 172-189, (Ochatt, S. and Jain, S.M., eds). Science Publishers, USA.

2. The Plant List (http://www.theplantlist.org)

3. Lim, T.K. (2015). *Edible Medicinal and Non-Medicinal Plants. Volume 9: Modified Stems, Roots, Bulbs*. New York: Springer.

4. O'Hair, S.K. and Maynard, D.N. (2003). Edible aroids. In: *Encyclopedia of Food Science and Nutrition*, PP. 5970-5973, (Trugo, L.C. Finglas, P.M. Belton, P. Ottaway, P.B., Bressani, R., *et al*., eds). Academic press, USA.
5. Giacometti, D.C. and Leon, J. (1994). Tannia, yautia (*Xanthosoma sagittifolium*). In: *Neglected Crops 1492 from Different Perspectives*, pp. 255–258, (Hernandez-Bermejo, L., ed). Food and Agricultural Organization of United the United Nations, Rome, Italy.
6. Govaerts, R., Frodin, D.G. and Bogner, J. (2002). World checklist and bibliography of Araceae (and Aroraceae). Royal Botanic Gardens, UK.

Reviewer #2

- **Design and Replication?**
  - Similar sized cormel germplasms were sampled from five Zones. Then, characterized using morphological traits by planting in a common research garden in **10x10 simple lattice square design for two years.** This is now clarified in the methodology.
  - The genotype by environmental interaction (GXE) evaluation is the next plan to be done. Therefore, multilocational trials is to be conducted in different agroecology to assess the magnitude of genotype x environment (GXE) interaction and to identify stable genotypes and evaluate their performance, selecting some genotypes from the clustered accessions to evaluated yield and yield related traits.

Reviewer #3

- **Data availability**
  - All data made to be available either as part of manuscript in table or as supplementary materials. This is also preferred comment from other two reviewers
- **Manuscript presentation**
  - Some improvements were made on the presentation of the paper as needed
- **Objective and Hypothesis**
  - Objective is clarified and hypothesis is elaborated
- **Sample collection zones**
  - The accessions that were collected from the same zone were considered as one population for the genetic diversity studies in assumption that cormel germplasms were more likely shared within zones than among zones. The same language is spoken within the zones and there are frequent local markets within zones where local people buy and sell commodities. **This is added in the methods and material section for clarity**

Results

- **3.1 Start the result section with clear finding**
  - Edited to meet the reviver's comment
- **3.2 Please don't start a sentences with a Table**
  - The comment is accepted and corrected
- **I would combine Table 2 and Table 3, emphasis to statistical significant character**
  - The comment accepted and the two tabled combined. Emphasis was given to statistical significant character, where statistical comparison was made at p= 0.05, 0.01 and 0.001
- **3.3 Please mention the link to Table 4 early in the text**
  - The link to table 4 is mentioned early in the text
- **Please check data and recalculate data for Ho =1**
  - Data checked and recalculated for Ho =1 for two loci (mXsCIR22 and mXsCIR27) (Table 3) and six SSR markers presented Ho values higher than He values. SSR markers, which we have used were initially identified by Cathebras et al. [1], gave variable degrees of heterozygosity, observed at levels ranging from 0.00 to 0.97. This excess in heterozygotes might be due to high rates of asexual reproduction in cocoyam due to vegetative propagation. The inflorescence of cocoyam is protogynous [2].

1. Catherbras C. Traore C. Malapa R. Risterucci A. Chair H. characterization of microsatellites in *Xanthosoma sagittifolium* (Araceae) and cross-amplification in related species. Appl Plant Sci. 2014; 2(6); 2(6):129-134 http://doi.org/10.3732/apps.1400027.
2. Manner HI. Farm and forestry production and marketing profile for Tannia (*Xanthosoma* spp.). In: Elevitch CR, editor. Specialty Crops for Pacific Island Agroforestry. Permanent Agriculture Resources, Hawaii, USA; 2011. pp. 1-16.

- **How many samples from each population**
    - Twelve to twenty eight accessions were sample from each zone. We clarified this now in the methodology section and the accessions passport data is presented as supplementary information (S1 Table 1) according the reviewer #1 and Reviewer #2 comments
- **3.4 Don't repeat the values in the Table, extract the most important findings**
    - Comment accepted accordingly
- **3.5 is not worth a separate chapter. Please combine with the previous**
    - Combined and the main finding is interpreted at the discussion section
- **3.6 Is this clustering of accessions separate from the morphological analysis?**
    - No, clusters were done by using both morphological and SSR markers data. The results were presented by Fig. 2 and Fig. 3 to see how one goes with the other. It was seen that the second larger cluster (C-III) was contained 27 (27%) of the total accessions. Of the 27 accessions grouped in this cluster, 25 (92.3%) were purple-cocoyam accessions (Fig 2). This result was obtained by using data of morphological genetic traits. Molecular data was also grouped almost all purple-cocoyam accessions in one cluster and the green ones in another cluster (Fig.2), supporting the morphological data. These results were presented in the result section and possible reasons for this were explained in the discussion section.
- **In M&M it was not explained why clustering seems to be important**
    - Checked and explained
- **How clustering was conducted**
    - The following sentences are in the M&M which explain how clustering of accession were conducted.
        - The quantitative morphological traits were subjected to a cluster analysis using Minitab17.1, which was conducted by employing average linkage clustering strategy of the observation. Variables were standardized to a common scale by subtracting the means and dividing by the standard deviation. The number of clusters was determined by following the steps recommended by Minitab 17.1. The dendrogram showing the Euclidean distance between clusters was constructed by plotting the results of cluster analysis using the same program.
        - The genetic clustering based on SSR data was assessed by performing using neighbour joining algorithm implemented in the computer program PHYLIP version 3.6 using Nei's genetic distance based on the frequencies generated by using MSA version 4.05.

Discussion

- **is too descriptive, several values that were mentioned in the result section were mentioned again, the discussion must be shortened, taxonomic conclusion need to be drawn**
- These comments were considered carefully and corrected accordingly.
