# Supplementary material for: Genetic diversity of Ethiopian cocoyam (Xanthosoma sagittifolium (L.) Schott) accessions as revealed by morphological traits and SSR markers
Source: PLoS One. 2021 Jan 7;16(1):e0245120. doi: 10.1371/journal.pone.0245120 (PMC7790241; doi:10.1371/journal.pone.0245120)
Supplement: Attachment — Submitted filename: Response to reviewers.docx [file pone.0245120.s005.docx]

**Response to Reviewers**

**First we would like to thank the reviewers for taking their time to give us these constructive comments. We have replied to the comments as follows:**

- **Response to general comments**
  - Our manuscript have been carefully checked and revised as needed according to the PLOS ONE’s style requirements.
  - The financial disclosure statement is changed and the cover letter is updated
  - Figure files are resubmitted carefully checking PLOS ONE requirements of figure files
  - ORCID included
  - Captions for supporting information files are included at the end of the manuscript and in text citations are updated

**Reply to reviewer’s comment**

**Reviewer #1**

- **Clarify objective and elaborate hypothesis.**
  - Objective is clarified and hypothesis is elaborated
- **Provide passport data?**
  - Provided as supplementary information (S1 Table. Passport data)
- **Provide Map?**
  - Provided
- **Improve Quality of dendograms and figures?**
  - Improved
- **A reader may be confused about our study materials and feel that *Colocasia* might have been included because of a sentence in the introduction explaining that in Ethiopia the two species are considered synonymously.**
  - We have edited the sentence and clarified it to avoid any confusion
- **Size and diameter of corm?**
  - The size and diameter of *Xanthosoma* corm is 10 to 15 cm in diameter but here the average diameter of corm recorded from 3 replication for two years experiment were 4.29 to 7.73 cm and the overall mean was 5.70 cm. Such difference may be due to:
    1) Cocoyam is a perennial plant, but for practical purposes, the corm and cormles were intentionally measured separately at the end of 12^th^ months after plantation (annually). This annual harvest could be the reason for the size and diameter reduction. Actually, we encountered larger sized corm at some fields where cocoyam grown as perennial crop when we were collecting germplasms, but which was not at research field (where it was cultivated annually).
    2) The soil type of the experimental site could be another reason.
- **One or several *Xanthosoma* species?**
  - In general, the division of the genus *Xanthosoma* into species has been difficult [1]. There is much confusion, discrepancies and uncertainties regarding the taxonomy of *Xanthosoma* at the species level. Various names have been used synonymously [2-3], the same plant being given more than one species name [4]. Thus, the name of *Xanthosoma sagittifolium* (L.) Schott has usually been given to the most cultivated members of this genus (Giacometti and [5-6]. In the course of this study, we refer to the accepted species status of *X. sagittifolium* in The Plant List [2] in the Araceae family [6], which does not tackle taxonomy in further detail.
  - There was no substantial evidence to say that there were different *Xanthosoma* species in Ethiopia although qualitative morphological traits and SSR markers clustered accession into two groups. We have a plan to do taxonomic study on *Xanthosoma* species in Ethiopia. Generally, the taxonomy of *Xanthosoma* needs to be reconstructed.

1. Saborio, F. (2007). Cocoyam (*Xanthosoma sagittifolium* (L.) Schott). In: *Breeding of Neglected and Underutilized Crops, Spices and Herbs, pp.* 172-189, (Ochatt, S. and Jain, S.M., eds). Science Publishers, USA.
2. The Plant List (<http://www.theplantlist.org>)
3. Lim, T.K. (2015). *Edible Medicinal and Non-Medicinal Plants. Volume 9: Modified Stems, Roots, Bulbs*. New York: Springer.
4. O’Hair, S.K. and Maynard, D.N. (2003). Edible aroids. In: *Encyclopedia of Food Science and Nutrition,* PP. 5970-5973, (Trugo, L.C. Finglas, P.M. Belton, P. Ottaway, P.B., Bressani, R., *et al*., eds). Academic press, USA.
5. Giacometti, D.C. and Leon, J. (1994). Tannia, yautia (*Xanthosoma sagittifolium*). In: *Neglected Crops 1492 from Different Perspectives*, pp. 255–258, (Hernandez-Bermejo, L., ed). Food and Agricultural Organization of United the United Nations, Rome, Italy.
6. Govaerts, R., Frodin, D.G. and Bogner, J. (2002). World checklist and bibliography of Araceae (and Aroraceae). Royal Botanic Gardens, UK.

**Reviewer #2**

- **Design and** **Replication?**
  - Similar sized cormel germplasms were sampled from five Zones. Then, characterized using morphological traits by planting in a common research garden in **10x10 simple lattice square design for two years.** This is now clarified in the methodology.
  - The genotype by environmental interaction (GXE) evaluation is the next plan to be done. Therefore, multilocational trials is to be conducted in different agroecology to assess the magnitude of genotype x environment (GXE) interaction and to identify stable genotypes and evaluate their performance, selecting some genotypes from the clustered accessions to evaluated yield and yield related traits.

**Reviewer #3**

- **Data availability**
  - All data made to be available either as part of manuscript in table or as supplementary materials. This is also preferred comment from other two reviewers
- **Manuscript presentation**
  - Some improvements were made on the presentation of the paper as needed
- **Objective and Hypothesis**
  - Objective is clarified and hypothesis is elaborated
- **Sample collection zones**
  - The accessions that were collected from the same zone were considered as one population for the genetic diversity studies in assumption that cormel germplasms were more likely shared within zones than among zones. The same language is spoken within the zones and there are frequent local markets within zones where local people buy and sell commodities. **This is added in the methods and material section for clarity**

**Results**

- **3.1 Start the result section with clear finding**
  - Edited to meet the reviver’s comment
- **3.2 Please don’t start a sentences with a Table**
  - The comment is accepted and corrected
- **I would combine Table 2 and Table 3, emphasis to statistical significant character**
  - The comment accepted and the two tabled combined. Emphasis was given to statistical significant character, where statistical comparison was made at p= 0.05, 0.01 and 0.001
- **3.3 Please mention the link to Table 4 early in the text**
  - The link to table 4 is mentioned early in the text
- **Please check data and recalculate data for Ho =1**
  - Data checked and recalculated for Ho =1 for two loci (mXsCIR22 and mXsCIR27) (Table 3) and six SSR markers presented Ho values higher than He values. SSR markers, which we have used were initially identified by Cathebras et al. [1], gave variable degrees of heterozygosity, observed at levels ranging from 0.00 to 0.97. This excess in heterozygotes might be due to high rates of asexual reproduction in cocoyam due to vegetative propagation. The inflorescence of cocoyam is protogynous [2].
    1. Catherbras C. Traore C. Malapa R. Risterucci A. Chair H. characterization of microsatellites in *Xanthosoma sagittifolium* (Araceae) and cross-amplification in related species. Appl Plant Sci. 2014; 2(6); 2(6):129-134 <http://doi.org/10.3732/apps.1400027>.
    2. Manner HI. Farm and forestry production and marketing profile for Tannia (*Xanthosoma* spp.). In: Elevitch CR, editor. Specialty Crops for Pacific Island Agroforestry. Permanent Agriculture Resources, Hawaii, USA; 2011. pp. 1-16.
- **How many samples from each population**
  - Twelve to twenty eight accessions were sample from each zone. We clarified this now in the methodology section and the accessions passport data is presented as supplementary information (S1 Table 1) according the reviewer #1 and Reviewer #2 comments
- **3.4 Don’t repeat the values in the Table, extract the most important findings**
  - Comment accepted accordingly
- **3.5 is not worth a separate chapter. Please combine with the previous**
  - Combined and the main finding is interpreted at the discussion section
- **3.6 Is this clustering of accessions separate from the morphological analysis?**
  - No, clusters were done by using both morphological and SSR markers data. The results were presented by Fig. 2 and Fig. 3 to see how one goes with the other. It was seen that the second larger cluster (C-III) was contained 27 (27%) of the total accessions. Of the 27 accessions grouped in this cluster, 25 (92.3%) were purple-cocoyam accessions (Fig 2). This result was obtained by using data of morphological genetic traits. Molecular data was also grouped almost all purple-cocoyam accessions in one cluster and the green ones in another cluster (Fig.2), supporting the morphological data. These results were presented in the result section and possible reasons for this were explained in the discussion section.
- **In M&M it was not explained why clustering seems to be important**
  - Checked and explained
- **How clustering was conducted**
  - The following sentences are in the M&M which explain how clustering of accession were conducted.
    - The quantitative morphological traits were subjected to a cluster analysis using Minitab17.1, which was conducted by employing average linkage clustering strategy of the observation. Variables were standardized to a common scale by subtracting the means and dividing by the standard deviation. The number of clusters was determined by following the steps recommended by Minitab 17.1. The dendrogram showing the Euclidean distance between clusters was constructed by plotting the results of cluster analysis using the same program.
    - The genetic clustering based on SSR data was assessed by performing using neighbour joining algorithm implemented in the computer program PHYLIP version 3.6 using Nei’s genetic distance based on the frequencies generated by using MSA version 4.05.

**Discussion**

- **is too descriptive, several values that were mentioned in the result section were mentioned again, the discussion must be shortened, taxonomic conclusion need to be drawn**
  - These comments were considered carefully and corrected accordingly.
