# Supplementary material for: Genetic diversity of Ethiopian cocoyam (Xanthosoma sagittifolium (L.) Schott) accessions as revealed by morphological traits and SSR markers
Source: PLoS One. 2021 Jan 7;16(1):e0245120. doi: 10.1371/journal.pone.0245120 (PMC7790241; doi:10.1371/journal.pone.0245120)
Supplement: Attachment — Submitted filename: Response to reviewers.docx [file pone.0245120.s007.docx]

**Response to Reviewers**

**First we would like to thank the reviewers again for taking their precious time to give us constructive comments. We have replied to the comments as follows:**

- **Reply to reviewer’s comment**
  - Our manuscript has been carefully checked per the PLOS ONE’s style requirements

**Reviewer #1**

1. **Data availability?**

- All available data, underlying the findings described in our manuscript, are provided as part of the manuscript and its supporting information files, without restriction.

2. **The authors should identify the number of multilocus genotypes (MLGs) and discuss the possible number of distinct clonal lineages before they attempt to conduct crosses.**
   - We have edited our conclusion and further plan according to this reviewer comment

**Reviewer #1 and Reviewer #2**

1. **There are language and writing errors**
   - We would like to thank reviewer #2 for the given corrections. We have accepted the corrections accordingly and further professionally editing was also done per the recommendation forwarded by reviewer #2.
